# Interfacial Microstructure and Tensile–Shear Properties of AA1060/SUS304 Dissimilar Sheet Joints Fabricated by Uniform-Pressure Electromagnetic Pulse Welding

**DOI:** 10.3390/ma19204266

**Published:** 2026-10-09

**Authors:** Zhisong Fan, Ying Huang, Shu Zhang, Jianghua Deng, Hanwei Ning, Minjie Huang

**Affiliations:** School of Mechanical Engineering and Automation, Fuzhou University, Fuzhou 350116, China; zhisongfan@fzu.edu.cn (Z.F.);

**Keywords:** dissimilar metal sheets, magnetic pulse welding, uniform-pressure electromagnetic actuator, interfacial microstructure, tensile–shear properties

## Abstract

Compared with single-turn coil magnetic pulse welding, magnetic pulse welding using a uniform-pressure electromagnetic actuator (UP-EMPW) can provide a larger area of uniform pressure and address issues such as low energy utilization efficiency and short coil lifespan. In this work, AA1060 aluminum sheets were joined to SUS304 stainless steel sheets by UP-EMPW. The effects of discharge voltage and initial gap on the interfacial microstructure and mechanical properties of the joints were investigated by optical microscopy, scanning electron microscopy (SEM), energy-dispersive spectroscopy (EDS), and tensile shear tests. Two distinct interface morphologies were observed: wavy interfaces and straight interfaces. Their formation was influenced by the combined effects of discharge voltage and initial gap. Microhardness measurements across the weld interface were further conducted to evaluate the local mechanical response and to help interpret the strengthening mechanisms associated with different interfacial features. The results showed that straight interfaces without a transition zone exhibited hardness values between those of the base materials, whereas wavy interfaces with a transition zone showed higher hardness values than both parent materials. Although these hardness data should be considered qualitative because of the limited indentation spacing, the overall trend suggests a stronger local strengthening effect at the wavy interface. The tensile shear tests indicated that the maximum load first increased and then decreased with increasing discharge voltage or initial gap. The highest joint performance within the investigated range was obtained at a discharge voltage of 9 kV and an initial gap of 1.5 mm, with a mean maximum tensile–shear load of 3288 ± 256 N and failure in the aluminum base material. The improved mechanical performance appeared to result from the combined effects of mechanical interlocking and metallurgical bonding associated with the wavy interface and transition zone. Fracture surface analysis further showed that base material fractures exhibited mixed ductile and brittle features, whereas pull-out failures were characterized by localized bonding and limited interfacial bonding.

## 1. Introduction

Lightweight design has become a central objective in many engineering fields, driven by the need to reduce energy consumption and emissions [1]. Multi-material hybrid structures, in which different materials are strategically combined, have emerged as a key approach to achieving this goal [2,3]. Among the candidate materials, aluminum alloys are particularly attractive because of their low density, high specific strength, and good corrosion resistance, making them suitable for partially replacing conventional steels in structural applications [4,5]. In practice, the integration of aluminum and steel can be realized through two distinct routes: one is the direct fabrication of heterogeneous composite plates via rolling, extrusion, or welding, which combines the two materials over a large area [6]; the other is the local joining of individual aluminum and steel sheets into lap configurations, which is particularly suitable for automotive body panels and similar structures where weight reduction is required without sacrificing local mechanical performance [7]. The latter route, however, still faces significant challenges in achieving reliable interfacial bonding.

Fusion welding processes, which are widely used for similar metal joints, are generally unsuitable for aluminum/steel dissimilar joints. The large differences in melting point, thermal conductivity, and metallurgical compatibility between aluminum and steel often lead to severe composition segregation, high residual stresses, and hot cracking in the joint [8]. In addition, excessive brittle intermetallic compounds (IMCs) tend to form at the interface, severely degrading the joint strength [9]. Alternative solid-state joining methods, such as friction stir welding [10], laser welding [11], and explosive welding [12], have been explored, but each has specific limitations in terms of equipment cost, joint configuration, or scalability.

Magnetic pulse welding (MPW), a solid-state impact welding technique, has attracted increasing attention for dissimilar metal joining [13]. In MPW, a pulsed electromagnetic force drives one workpiece to collide with another at an extremely high speed, creating metallurgical bonding in the solid state. Because the process is extremely fast and involves limited heat input, the excessive formation of brittle IMCs can be effectively suppressed [14]. MPW has been successfully applied to various dissimilar metal combinations, including Al-Cu, Al-Mg, and Al-Fe [15]. MPW shares many characteristics with explosive welding, as both are high-speed impact welding processes. In both cases, the workpieces collide at high velocity, leading to severe plastic deformation, localized melting, and rapid solidification at the interface. As a result, wavy interfaces and IMC layers are commonly observed in both processes [16,17]. Extensive studies on explosive welding have shown that the thickness and morphology of the IMC layer strongly influence the joint strength: a thick and continuous IMC layer often leads to brittleness and crack initiation [18,19,20,21]. These findings provide useful guidance for understanding the interfacial phenomena in MPW. However, unlike explosive welding, MPW is driven by electromagnetic force, does not require explosives, and offers better controllability and repeatability, making it particularly suitable for sheet lap joining in lightweight structures [22].

Depending on the workpiece geometry, MPW can be classified into tube welding and sheet welding. Tube welding is relatively mature and has been widely applied in industry [23]. Sheet welding, however, is still in an early stage of development. Most existing sheet MPW systems employ single-turn coils, which suffer from low energy utilization efficiency, non-uniform magnetic field distribution, inconsistent weld formation, and short coil service life [24]. These shortcomings limit the scalability of sheet MPW for industrial production.

To overcome these limitations, researchers have explored alternative coil structures. Sarvari et al. [25] developed a double-sided H-shaped coil for joining Al1050 to copper. Berlin et al. [26] employed a double-sided H-shaped coil for lap joints of AZ31 Mg alloy sheets. Aizawa et al. [27] designed a one-layer E-shaped flat coil for joining aluminum alloys to SPCC steel sheets, which improved weld quality and could be adapted to workpieces of different sizes. Wang et al. [28] employed a one-layer E-shaped coil to join AA1060 to AZ31B magnesium alloy. More recently, Li et al. [29] proposed a multi-seam coil for Al/Ti plate magnetic pulse welding; this coil configuration effectively modulates the Lorentz force distribution on the flyer plate and provides a larger effective welding area. Despite these improvements, single-turn or multi-arm flat coils still face challenges in achieving uniform pressure distribution and sufficient coil durability, which remain critical issues for sheet MPW.

The uniform-pressure electromagnetic actuator (UPEA), which uses a multi-turn coil and provides a more uniform pressure distribution, has been identified as a promising solution [30]. Briefly, a capacitor bank discharges through a multi-turn spiral coil, generating a pulsed magnetic field. This magnetic field induces eddy currents in a closed secondary circuit formed by the conductive channel and the flyer plate. The induced current flows in the opposite direction to the coil current, producing a repulsive Lorentz force. This force is transmitted uniformly to the flyer plate, accelerating it to a high velocity and causing it to collide with the target plate, thereby achieving bonding. Weddeling et al. [31] first demonstrated the feasibility of using a UPEA for sheet MPW and proposed a simplified analytical model to predict workpiece velocity. On this basis, Yu and Tong [32] successfully welded AA1060 aluminum to Q235 steel using a UPEA and established a preliminary weldability window. More recent studies have focused on optimizing the UPEA design and welding procedure to enlarge the weld area and suppress defects such as center bulging and flyer thinning at the chamfer [33,34,35,36]. For example, a pre-deformation method was proposed to control the central impact angle, which effectively suppressed center non-welding and bulging [35]. Numerical simulations have also been performed to analyze the deformation process and optimize the actuator geometry [36].

Despite these advances, existing studies have mainly concentrated on feasibility validation, actuator design optimization, and defect suppression in UPEA-based MPW. A deep understanding of how the key process parameters, especially discharge voltage and initial gap, jointly influence the interfacial microstructure evolution (wavy/straight interfaces, transition zone formation, base element distribution) and the resulting tensile–shear properties is still lacking. In particular, the relationship between interfacial characteristics and mechanical performance of AA1060/SUS304 dissimilar sheet joints has not been clearly established.

In this study, uniform-pressure electromagnetic pulse welding was employed to join AA1060 aluminum sheets to SUS304 stainless steel sheets. The effects of discharge voltage and initial gap on the macro-morphology, interfacial microstructure, and mechanical properties of the joints were systematically investigated through tensile–shear tests, microhardness measurements, and SEM/EDS characterization. The aim is to reveal the process-microstructure-performance correlation and to provide an experimental basis for optimizing the UPEA-based MPW process for aluminum/steel dissimilar sheet joining.

## 2. Experimental Materials and Methods

### 2.1. Experimental Principle and Equipment

**Figure 1:**
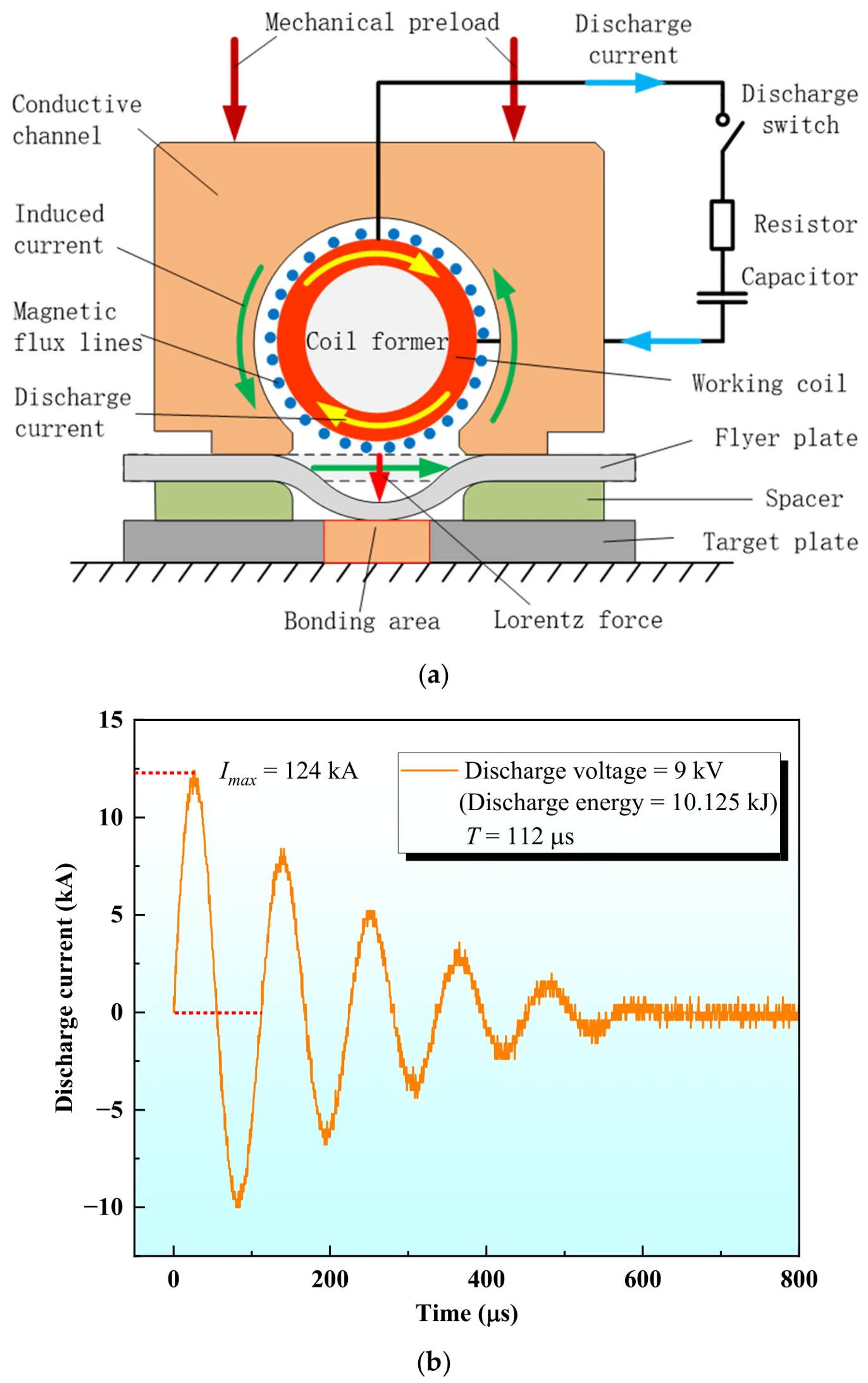
(**a**) Schematic illustration of the uniform-pressure electromagnetic pulse welding (UP-EMPW) process and (**b**) the typical discharge current waveform at a discharge voltage of 9 kV.

The working principle of the uniform-pressure electromagnetic pulse welding (UP-EMPW) process used in this study is illustrated in Figure 1a. From the inside to the outside, the system comprises a spiral coil, a conductive channel, a flyer plate, a spacer, and a target plate. During welding, the conductive channel and the flyer plate are pressed together to form a closed secondary circuit. The discharge current in the spiral coil induces an eddy current in the secondary circuit, and the interaction between the two currents generates a repulsive Lorentz force that drives the flyer plate to accelerate and collide with the target plate at high speed. When the collision speed and angle are within an appropriate range, bonding is achieved between the two plates.

The welding experiments were performed using an EMF-50/20 magnetic pulse welding system (Harbin Insititute of Technoligy, Harbin, China). The total capacitance of the capacitor bank is 250 μF; the rated charging voltage is 20 kV; and the rated discharge energy is 50 kJ. The discharge voltage in this work was adjusted according to different experimental requirements, covering a setting range from 8 kV to 11 kV. The discharge energy was calculated as *E* = 1/2*CU*^2^, where *C* = 250 μF is the capacitance of the capacitor bank, and *U* is the discharge voltage. For the investigated voltages, the corresponding energies were 8.0, 10.125, 12.5, and 15.125 kJ at 8, 9, 10, and 11 kV, respectively. The typical discharge current waveform, obtained at a discharge voltage of 9 kV, is shown in Figure 1b. The current exhibits a damped oscillatory waveform typical of an RLC circuit, with a first peak current of approximately 124 kA and an oscillation period of 112 μs.

**Figure 2:**
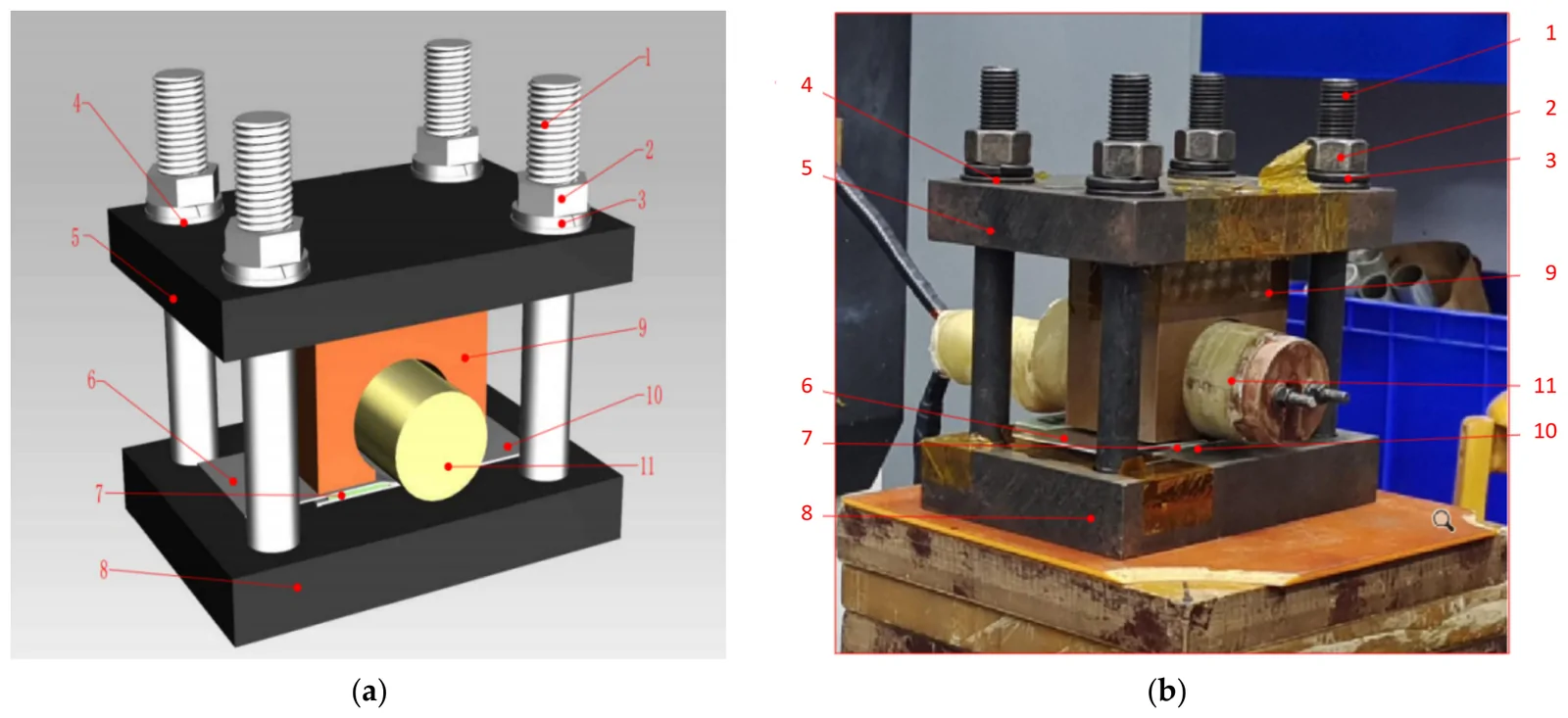
Welding apparatus used in the UP-EMPW experiments: (**a**) three-dimensional model and (**b**) photograph. Labels: 1, bolt; 2, nut; 3, spring washer; 4, flat washer; 5, cover plate; 6, flyer plate; 7, epoxy spacer; 8, base plate; 9, conductive channel; 10, target plate; 11, working coil.

The welding apparatus used in this study is shown in Figure 2. It consists of an external conductive channel, a seven-turn spiral coil, an epoxy resin plate, a base plate, a pressing plate, and several bolts and nuts. The working coil was wound from rectangular copper wire with a cross-section of 7 mm × 5 mm. It consisted of 7 turns. The conductive channel had a length of 80 mm, a width of 50 mm, and a height of 68 mm. The hole axis was located 25.5 mm from the lower surface of the channel, and the hole diameter was 49.5 mm. The lower end of the external conductive channel is in contact with the aluminum flyer plate to form a secondary circuit. A sufficiently large mechanical preload is applied to ensure reliable contact between the conductive channel and the flyer plate during discharge, thereby preventing arcing. The epoxy resin plate provides an acceleration distance for the flyer plate and incorporates chamfers to avoid excessive thinning and fracture of the aluminum sheet.

### 2.2. Experimental Materials

The base materials used in this study were commercially available AA1060 aluminum alloy and SUS304 stainless steel sheets (standard grade, purchased from a local supplier in China), both with dimensions of 100 mm × 50 mm. The aluminum sheet, used as the flyer plate, had a thickness of 0.8 mm, while the stainless steel sheet, used as the target plate, had a thickness of 1.0 mm. AA1060 aluminum alloy has a face-centered cubic structure, good electrical conductivity, and excellent plasticity, which satisfies the requirements for the flyer plate in magnetic pulse welding. SUS304 stainless steel was selected because of its high corrosion resistance and good mechanical properties under high-temperature and high-pressure conditions. Both base materials were supplied in the annealed (O-temper) condition. The nominal chemical compositions of the two materials are listed in Table 1 and Table 2, and their typical mechanical properties are summarized in Table 3. Before welding, the surfaces of both plates were cleaned with anhydrous ethanol to remove oil and other contaminants. The sheets were assembled in a lap joint configuration, with the aluminum sheet placed as the flyer and the stainless steel sheet as the target.

### 2.3. Mechanical Testing and Microstructural Characterization

**Figure 3:**
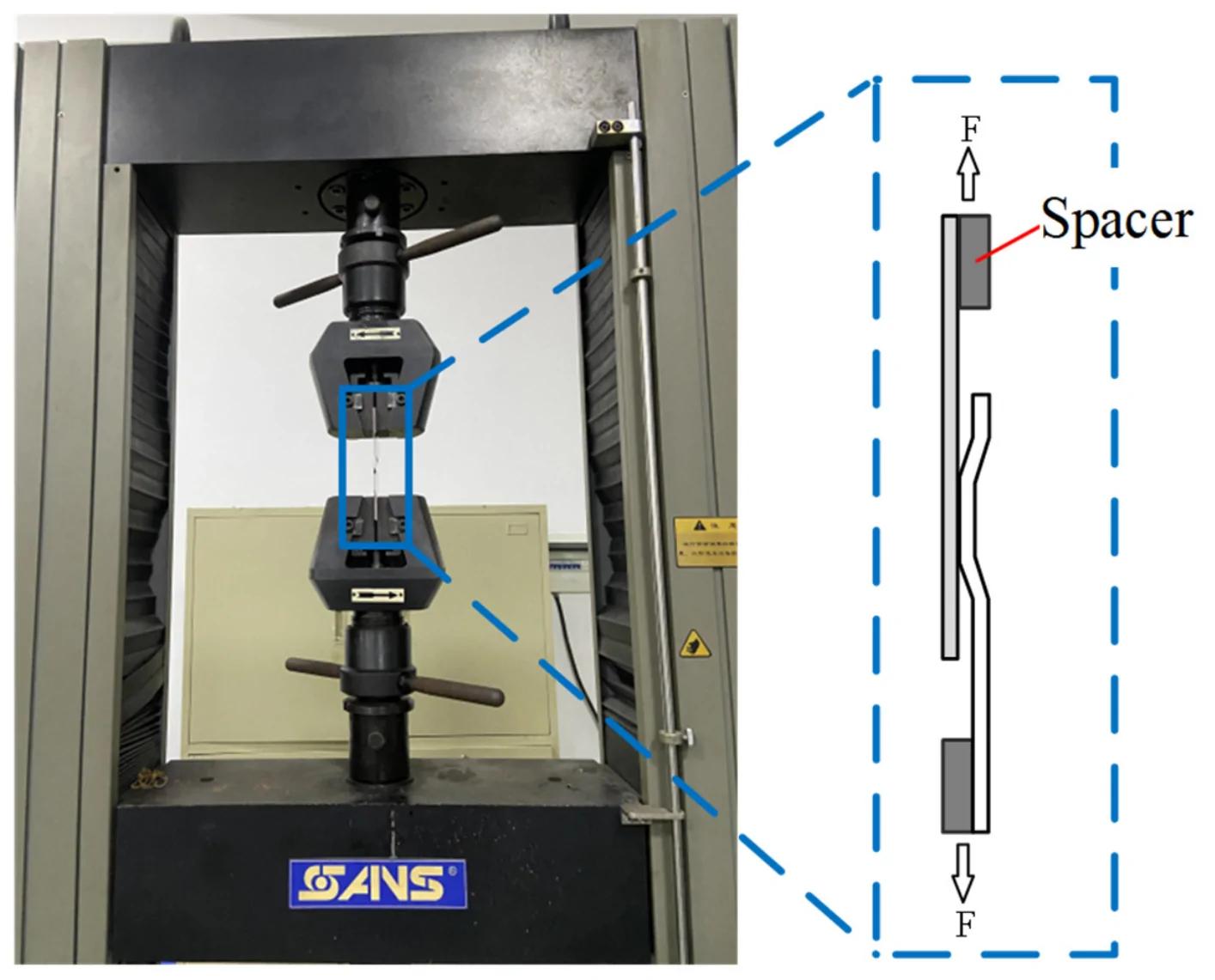
Schematic diagram of the tensile–shear test setup with spacer plates.

To evaluate the mechanical properties of the welded joints, tensile–shear tests were conducted at room temperature on a CMT5305 electronic universal testing machine (MTS Systems (China) Co., Ltd., Shanghai, China) at a crosshead speed of 1 mm/min. Three specimens were tested for each set of welding parameters. To ensure alignment and avoid eccentric loading, spacers of suitable thickness were placed on both sides of the lap joint, as schematically shown in Figure 3. For each set of welding parameters, three specimens were tested. The maximum tensile–shear load of each specimen was determined from the load–displacement curve, and the mean value and standard deviation were calculated. After testing, the fracture surfaces were examined using scanning electron microscopy(FEI Czech Republic s.r.o., Brno, Czech Republic).

Microhardness measurements were performed using an HVS-1000A digital microhardness tester (Laizhou Huayin Testing Instrument Co., Ltd., Laizhou, China) with a Vickers indenter. The purpose of these measurements was to evaluate the local mechanical response across the weld interface and to compare the strengthening effects of wavy and straight interfaces. The tests were carried out under a load of 0.98 N with a dwell time of 15 s. Hardness indentations were made along lines perpendicular to the weld interface. Three hardness profiles were measured perpendicular to the weld interface at three different positions. The mean value and standard deviation were calculated from the three measurements.

To observe the weld interface, specimens were cut perpendicular to the weld seam using wire electrical discharge machining. The cut specimens were cleaned with anhydrous ethanol, mounted in epoxy resin, and then ground with a series of SiC abrasive papers from low to high grit until the surface scratches were uniform. Polishing was performed using diamond paste followed by an alumina suspension to obtain a mirror-like finish. Because of the significant difference in hardness between aluminum and steel, the polishing procedure was carefully controlled to avoid excessive surface relief. The polished specimens were cleaned ultrasonically, rinsed with ethanol, and dried for subsequent examination. The interfacial morphology was observed using an FEI Quanta250 scanning electron microscope (FEI Czech Republic s.r.o., Brno, Czech Republic), and the elemental distribution at the interface was characterized by energy-dispersive spectrometry.

## 3. Results and Discussion

### 3.1. Macroscopic Appearance of the Joint

**Figure 4:**
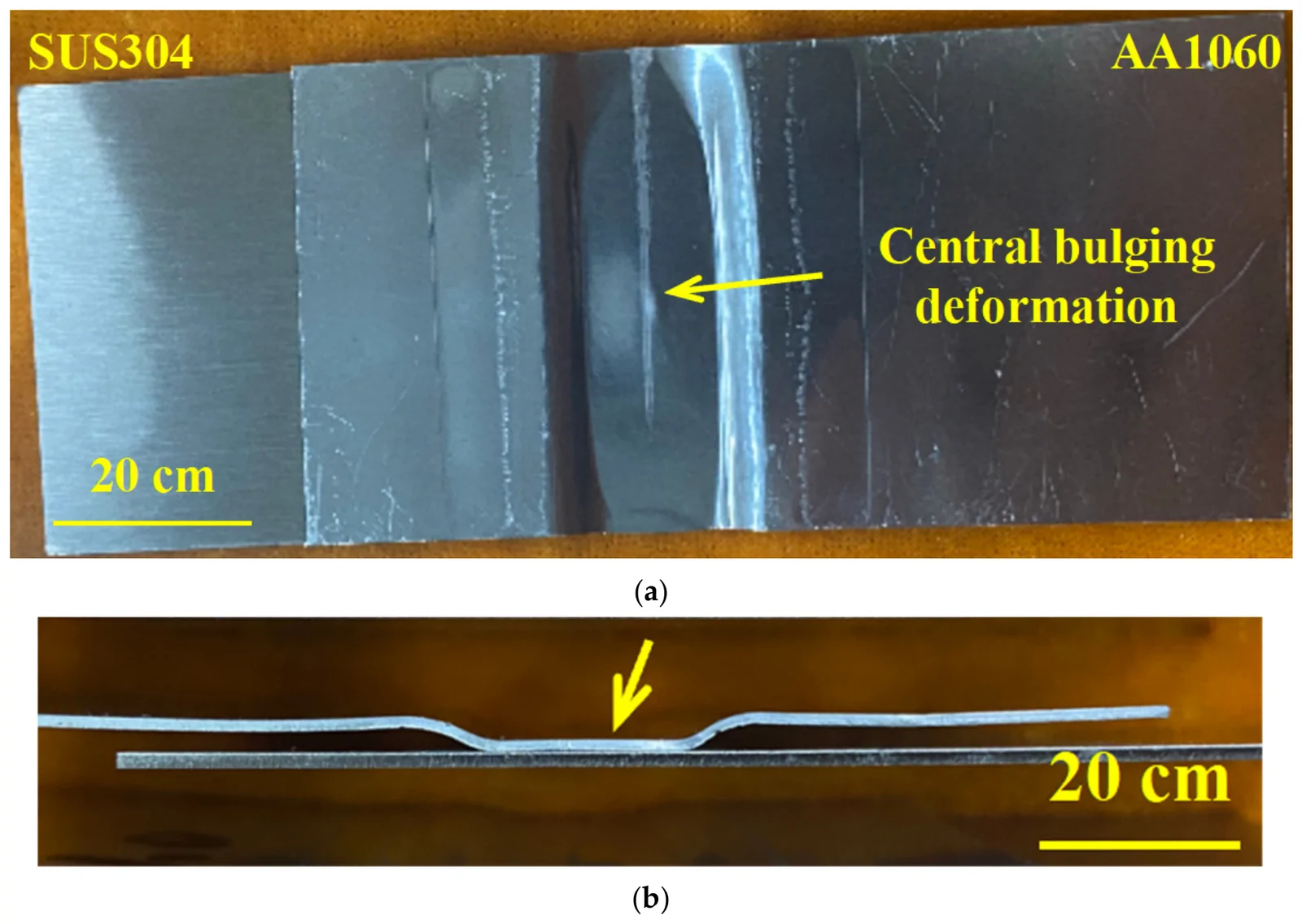
Typical macroscopic morphology of an AA1060/SUS304 joint: (**a**) top view and (**b**) side view.

**Figure 5:**
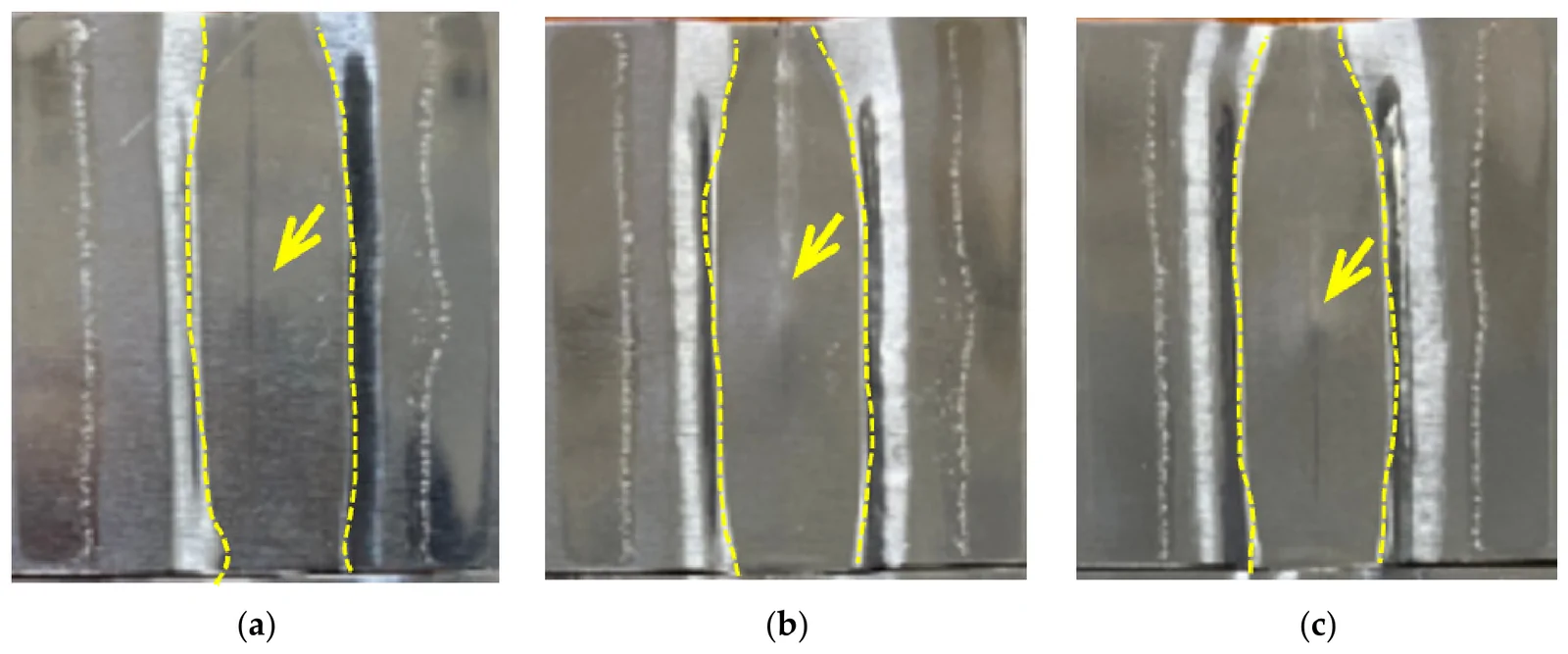
Macroscopic morphologies of joints welded under initial gap of (**a**) 1.0 mm, (**b**) 1.5 mm, and (**c**) 2.0 mm.

Figure 4 presents the typical macroscopic morphology of an AA1060/SUS304 joint obtained at an initial gap of 1.5 mm. A multi-square annular weld trace is clearly visible on the aluminum sheet surface, and a visible bulge is formed at the center of the weld zone. Similar multi-square annular morphologies were observed for all process parameters, as shown in Figure 5, although the degree of central bulging varied with initial gap. The formation of the central bulge can be attributed to two main factors. First, under the uniform-pressure electromagnetic actuator, the central region of the flyer does not achieve an effective collision angle with the target plate, resulting in a large rebound after impact. Second, because of the relatively large welding area, air trapped at the interface cannot be expelled sufficiently during the extremely short collision process, which also promotes the formation of a central bulge. This interpretation is supported by the finite element simulation results [33,35,36]. The simulation indicates that the central uniform-pressure region impacts the target almost parallel, causing significant rebound, while the collision propagates toward the edges with a certain angle, leading to the observed multi-square annular weld pattern.

**Figure 6:**
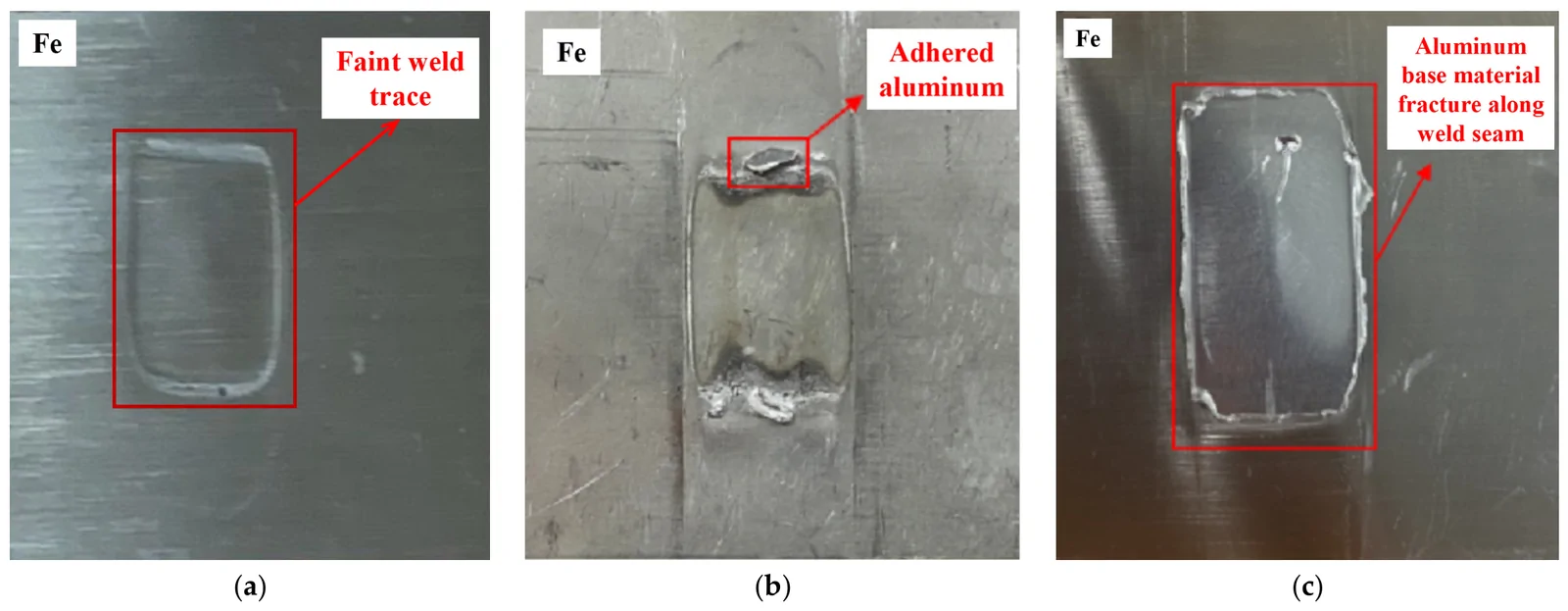
Representative peeled specimens showing three connection states: (**a**) unbonded, (**b**) partially bonded, (**c**) well bonded.

To preliminarily evaluate the joint connection quality before tensile–shear testing, peeling tests were conducted on specimens welded under different discharge voltages. Representative peeled specimens are shown in Figure 6. Three distinct connection states were identified: (i) unbonded joints, which separated freely or under very small force, leaving only a faint weld trace; (ii) partially bonded joints, in which a small amount of aluminum adhered to the stainless steel surface but the joint strength was lower than that of the weaker base material; and (iii) well-bonded joints, which fractured along the aluminum base material outside the weld zone. These qualitative observations provide a useful classification for interpreting the tensile–shear results presented in the next section.

### 3.2. Tensile–Shear Performance and Failure Modes

#### 3.2.1. Influence of Discharge Voltage

**Figure 7:**
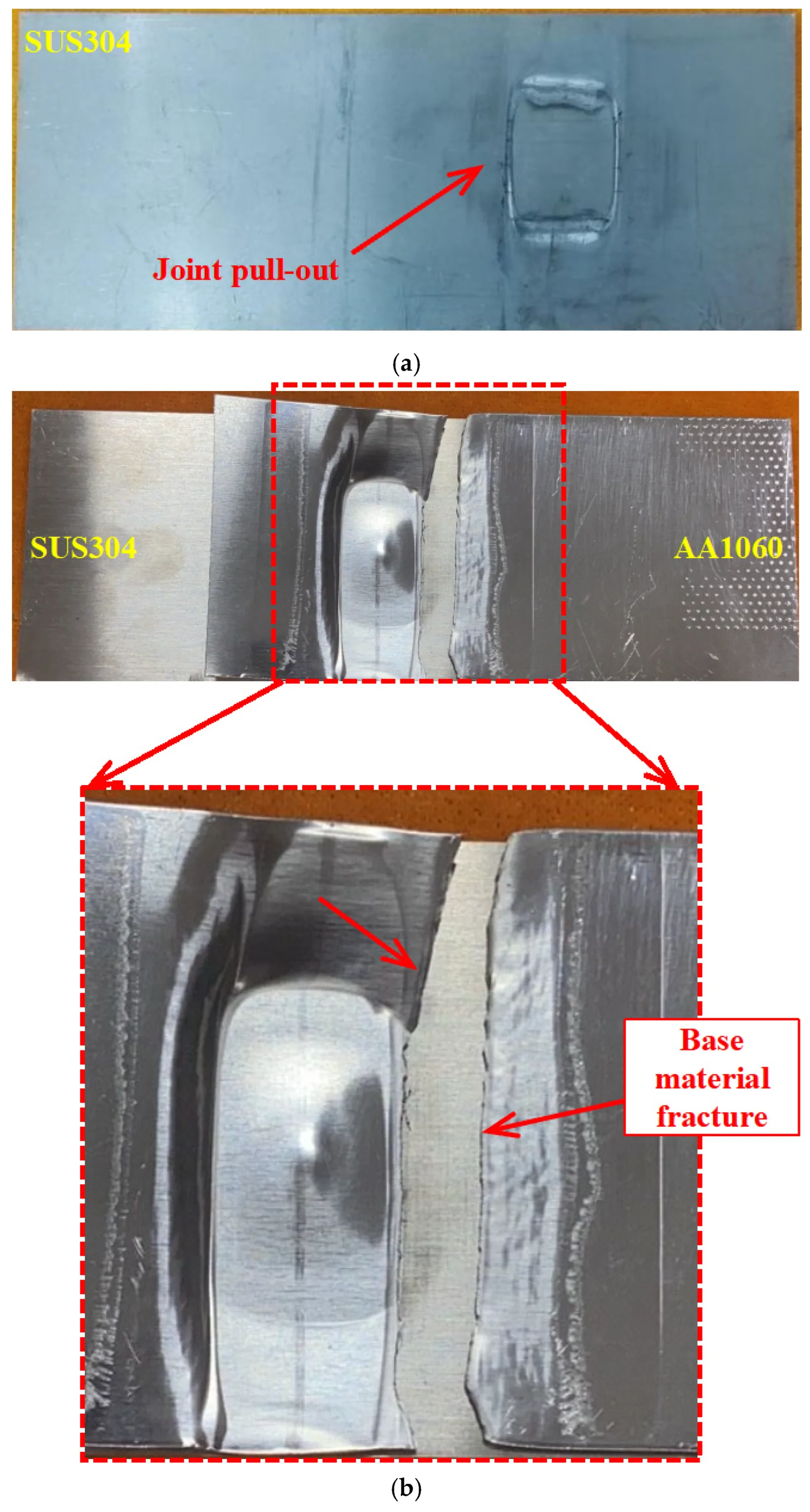
Representative failure modes of the welded joints after tensile–shear testing: (**a**) base material fracture and (**b**) joint pull-out.

**Figure 8:**
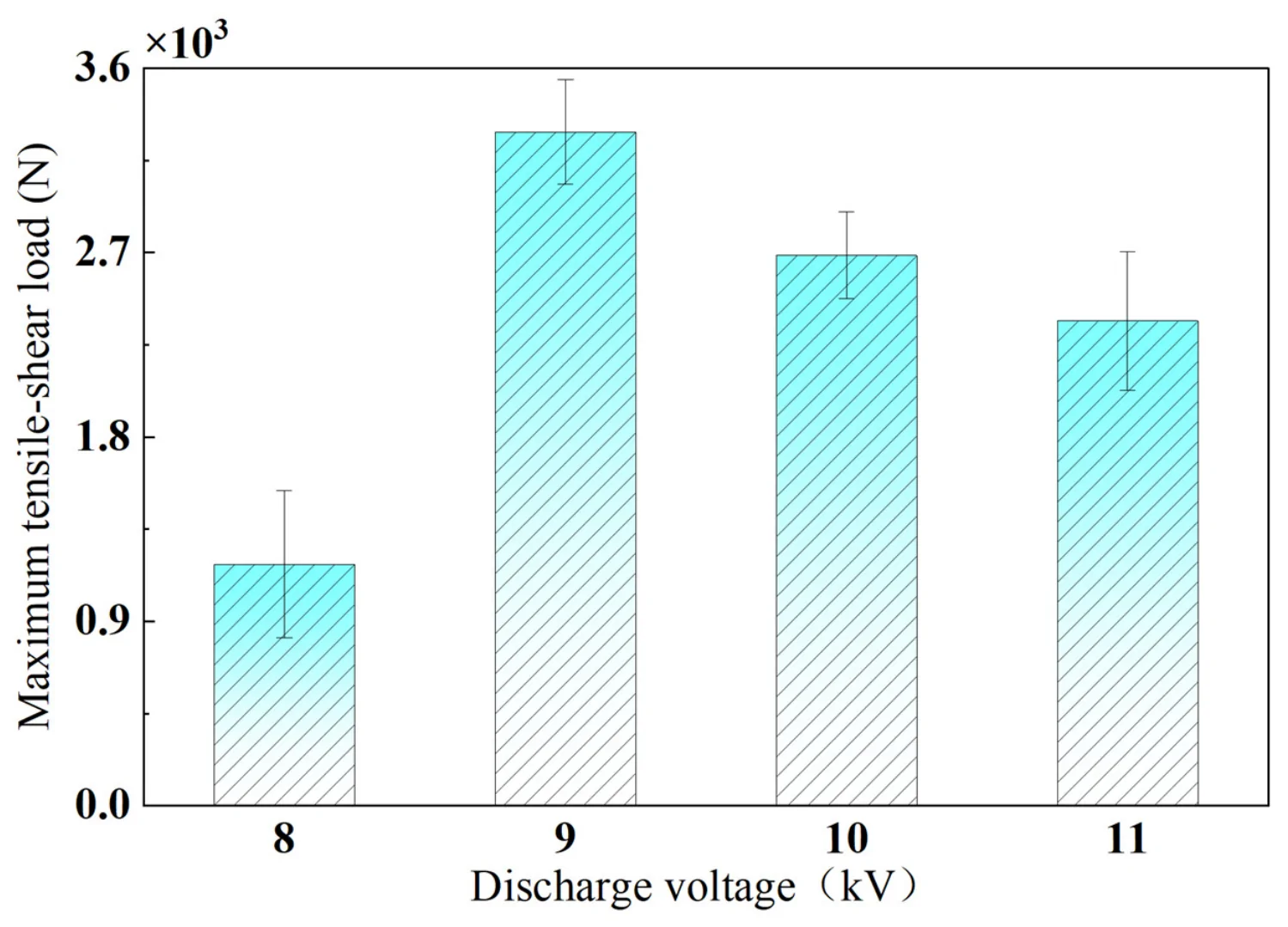
Maximum tensile–shear loads of joints welded at different discharge voltages (initial gap: 1.5 mm). Data are presented as mean ± standard deviation (*n* = 3).

To investigate the effect of discharge voltage on joint performance, tensile–shear tests were carried out on specimens welded at a fixed initial gap of 1.5 mm with discharge voltages of 8 kV, 9 kV, 10 kV and 11 kV. Two distinct failure modes were observed in the tensile–shear tests: base material fracture and joint pull-out, as illustrated in Figure 7. The maximum tensile–shear loads and corresponding failure modes are listed in Table 4, and the mean maximum tensile–shear loads with standard deviations are shown in Figure 8. The mean load increased from 8 kV to 9 kV, reaching a maximum of 3288 ± 256 N at 9 kV, and then decreased at 10 kV and 11 kV. This non-monotonic trend indicates that the highest joint performance within the investigated range was obtained at 9 kV.

In terms of failure mode, joints welded at 8 kV failed by joint pull-out, whereas those welded at 9 kV and above exhibited a shift to aluminum base material fracture. The improvement in joint strength from 8 kV to 9 kV may be attributed to the higher flyer collision speed, which promotes more effective bonding at the interface. However, excessively high voltage also intensifies plastic deformation of the aluminum flyer and, under the action of the preload and the chamfer geometry, leads to thinning of the flyer. This thinning effect counteracts the benefit of improved bonding and may eventually cause a decrease in the measured load-bearing capacity, as observed at 10 kV and 11 kV.

It should be noted that the error bars in Figure 8 represent one standard deviation of three measurements (*n* = 3). The 11 kV condition was included to examine the joint performance under higher energy input. Even under the same nominal parameters, the measured loads show some scatter. This scatter is most pronounced near the boundaries of the process window, i.e., at 8 kV and 11 kV. Because the local collision conditions were not directly measured in this work, the exact cause of this scatter cannot be fully identified. Possible reasons include slight specimen-to-specimen differences in the contact state and the actual gap after clamping. The limited number of specimens (*n* = 3) also contributes to the apparent scatter. Despite this, the overall trend is clear and consistent with the observed failure modes.

#### 3.2.2. Influence of Initial Gap Between Joining Surfaces

**Figure 9:**
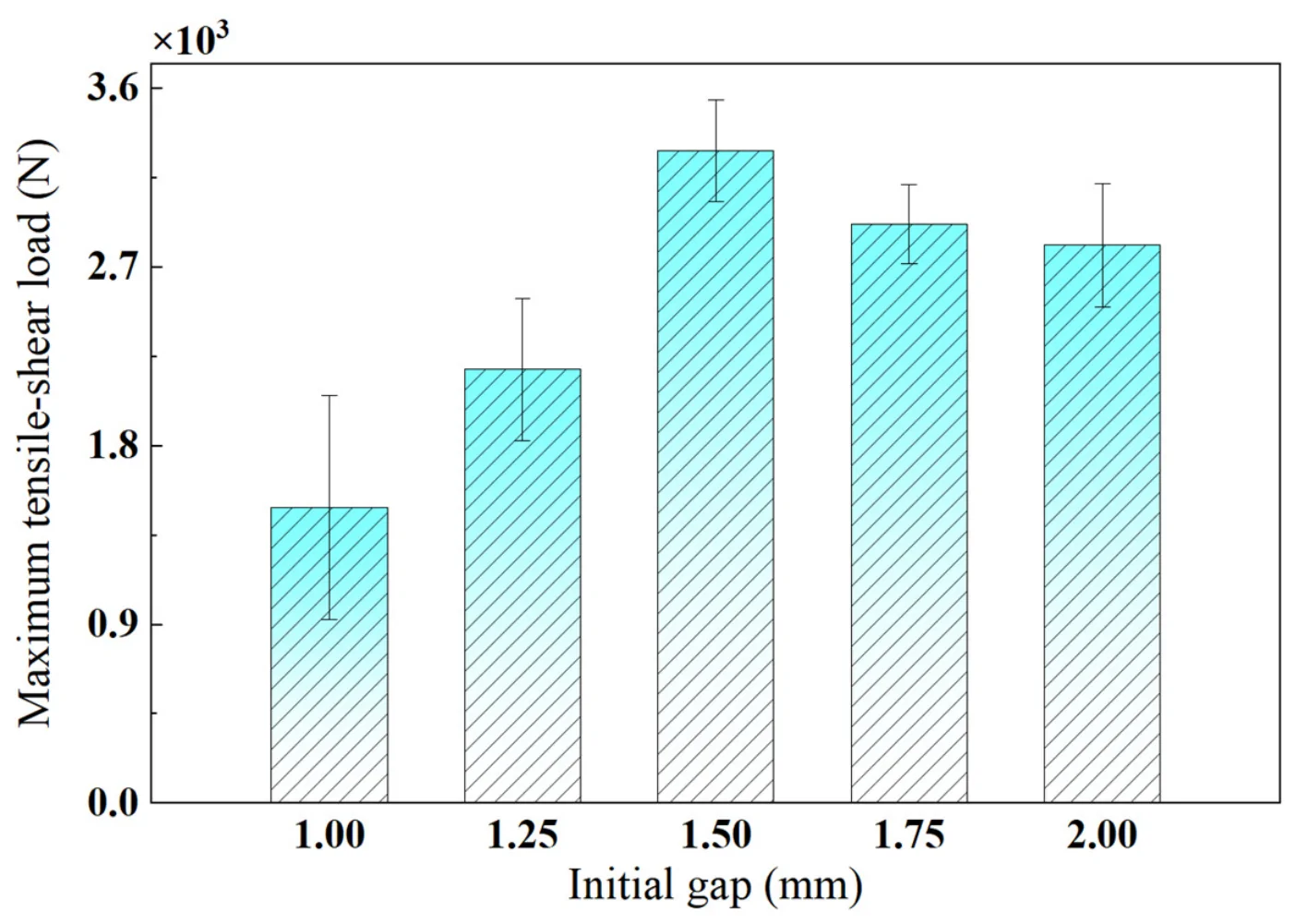
Maximum tensile–shear loads of joints welded at different initial gaps (discharge voltage: 9.0 kV). Data are presented as mean ± standard deviation (*n* = 3).

The influence of the initial gap was studied by keeping the discharge voltage constant at 9 kV and varying the gap from 1.0 mm to 2.0 mm. The maximum tensile–shear loads and failure modes are summarized in Table 5. The mean maximum tensile–shear loads and the corresponding standard deviations are plotted in Figure 9. As the initial gap increased from 1.0 mm to 1.5 mm, the mean load increased markedly, and the failure mode changed from joint pull-out to base material fracture. The mean maximum tensile–shear load at 1.5 mm was 3288 ± 256 N. Further increasing the gap from 1.5 mm to 2.0 mm resulted in a slight decrease in the mean maximum load, although the failure mode remained base material fracture.

These results suggest that a sufficient initial gap is required for the flyer to acquire adequate kinetic energy and an appropriate collision angle before impact. When the gap is too small, the acceleration distance is insufficient, and the flyer may not reach the velocity needed for effective bonding. Conversely, when the gap is too large, the flyer undergoes excessive plastic deformation during acceleration, leading to thinning and a reduction in the effective load-bearing area, which may slightly lower the joint strength. Under the present experimental conditions, the highest joint performance within the investigated range was obtained at an initial gap of 1.5 mm, at which a mean maximum tensile–shear load of 3288 ± 256 N was achieved and all specimens fractured in the aluminum base material.

Similar scatter is observed for specimens welded under the same nominal parameters. The scatter is pronounced at small gaps (1.0 mm and 1.25 mm). As noted in Section 3.2.1, the local collision conditions were not directly measured, and the scatter is likely related to slight differences in the actual gap after clamping, the contact state, and the local preload distribution. Although the sample size was limited to three specimens per condition, the overall trend is clear.

### 3.3. Preliminary Welding Process Window

**Figure 10:**
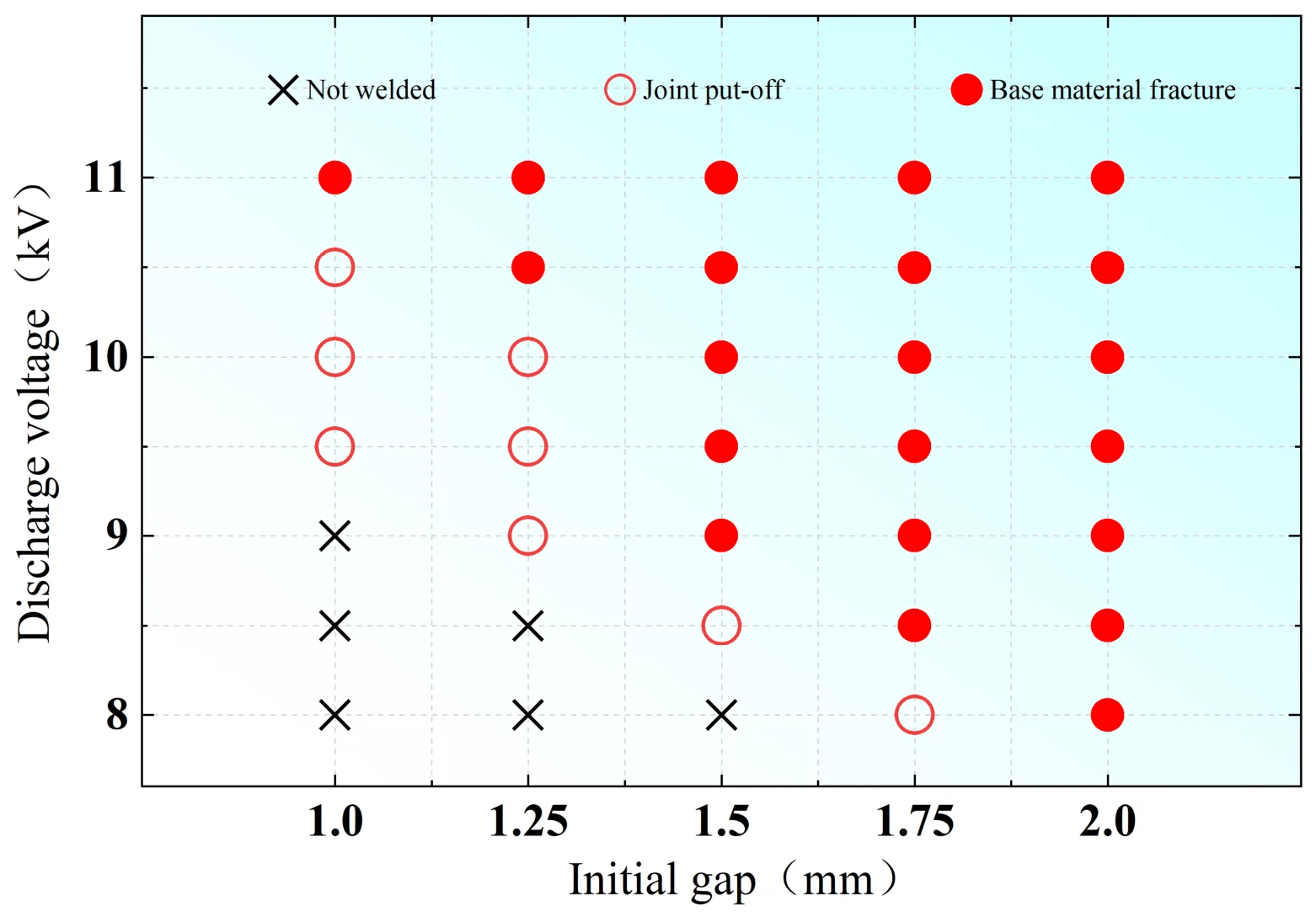
Preliminary welding process window for uniform-pressure electromagnetic pulse welding of AA1060/SUS304 sheets. The welding states are classified as follows: unbonded (specimens separated immediately after welding); partially bonded (joint pull-out with visible aluminum adhesion on the stainless steel surface); and well bonded (base material fracture). The boundaries are approximate due to the limited number of tested conditions.

Based on the tensile–shear test results and corresponding failure modes, a preliminary welding process window for uniform-pressure electromagnetic pulse welding of AA1060 to SUS304 was constructed from the parameter combinations investigated in this study, as shown in Figure 10. In this diagram, the horizontal axis represents the initial gap and the vertical axis represents the discharge voltage. The welding states are classified according to the post-welding condition and the tensile–shear failure mode. Specifically, unbonded specimens separated immediately after welding and could not be subjected to tensile–shear testing, corresponding to no measurable load-bearing capacity. Partially bonded joints survived handling and could be tested but failed by joint pull-out; in these specimens, aluminum adhesion was clearly visible on the stainless steel surface, indicating that localized bonding had occurred and that the joint possessed a certain load-bearing capacity. Well-bonded joints failed by base material fracture.

The results indicate that, within the investigated parameter range, increasing the initial gap generally facilitates the formation of a sound joint at a fixed discharge voltage, while at a fixed initial gap, a higher discharge voltage provides a larger discharge energy and therefore also tends to improve weldability. However, an appropriate match between discharge voltage and initial gap is important. When the initial gap is less than approximately 1.5 mm, the acceleration distance is too short, and the acceleration distance may be insufficient for the flyer to acquire sufficient kinetic energy before impact; consequently, a higher voltage is required to achieve acceptable bonding. On the other hand, when the initial gap exceeds about 2.0 mm, the flyer may undergo thinning or tearing at the chamfer, which may reduce the joint mechanical properties despite the higher discharge energy. In the present work, a high-quality joint was obtained at an initial gap of 1.5 mm and a discharge voltage of 9 kV, with all specimens failing in the aluminum base material. Therefore, the most favorable region within the investigated range was located around an initial gap of 1.5–2.0 mm combined with a moderate discharge voltage, where favorable bonding and flyer integrity were observed. Because only a limited number of parameter combinations were tested, the boundaries of this preliminary process window should be regarded as approximate, and further experiments are needed to refine them.

### 3.4. Microstructure of the Welded Interface

#### 3.4.1. Interfacial Morphology and Defect Analysis

**Figure 11:**
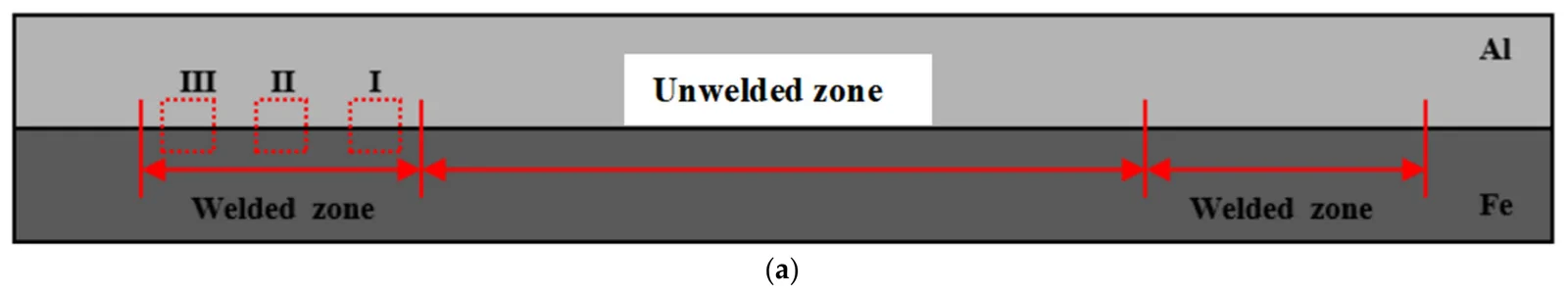

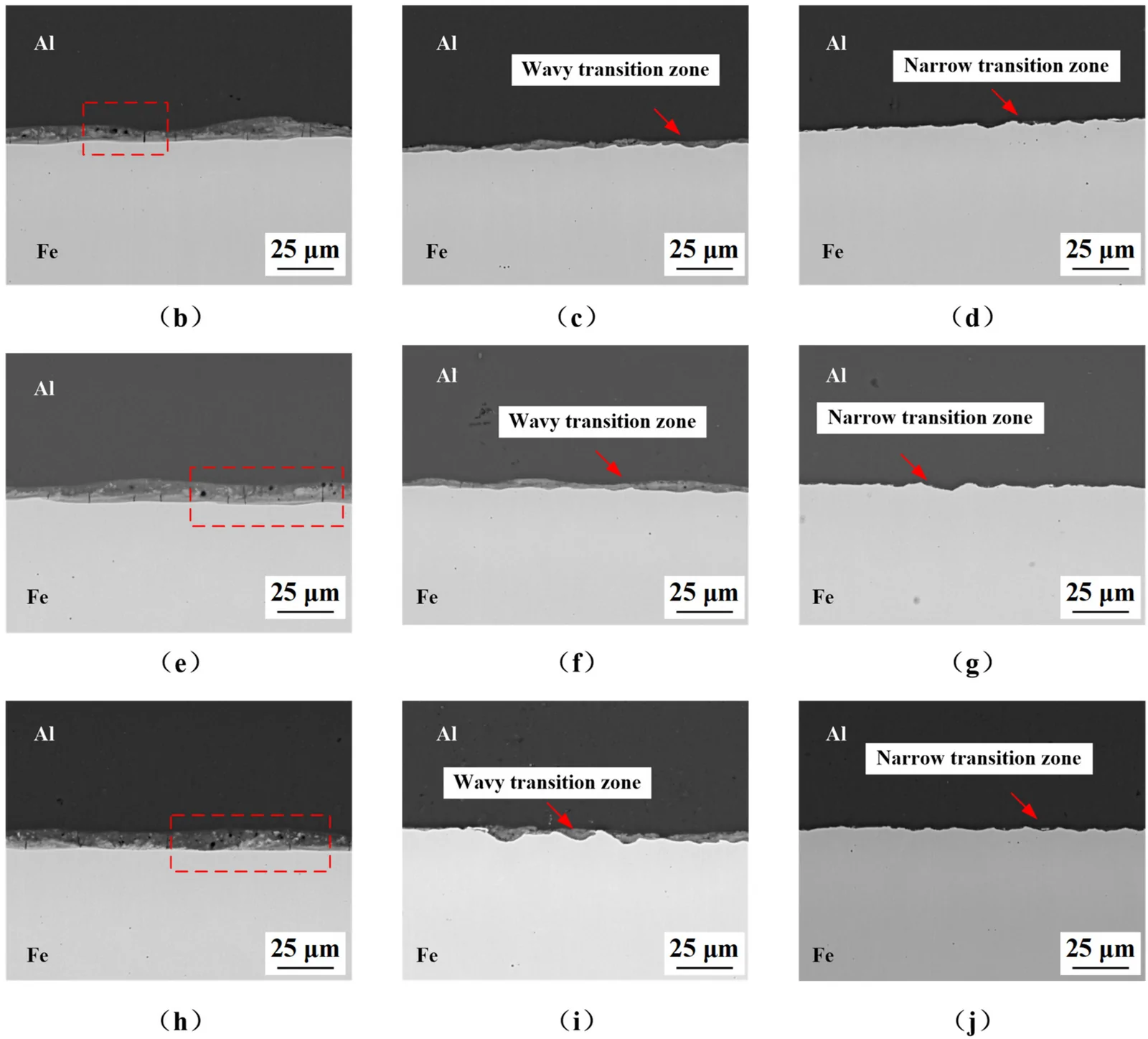
Interfacial morphology of a typical joint: (**a**) schematic cross-sectional view of the weld interface showing representative locations I, II, and III; (**b**–**j**) SEM images of the interface at locations I, II, and III for discharge voltages of 9 kV (**b**–**d**), 10 kV (**e**–**g**), and 11 kV (**h**–**j**) while keeping the initial gap constant at 1.5 mm.

To reveal the microstructural evolution at the welding interface, SEM observations were performed on joints welded under different process parameters. Figure 11a shows the overall cross-sectional view of a typical joint, which can be divided into a welded zone and an unwelded zone. Three representative locations, marked as I, II, and III, were selected along the weld interface for detailed examination. Position I is located near the center of the joint, adjacent to the unwelded zone. As shown in Figure 11b,e,h, at this position the interface exhibited a mixed structure of cracks and pores under all investigated discharge voltages (9 kV, 10 kV, and 11 kV). These defects are generally associated with poor mechanical performance, and such defect-containing regions typically extended over a width of approximately 7–10 μm along the interface, as estimated from the SEM images. Position II is located in the middle of the welded zone. The corresponding micrographs in Figure 11c,f,i show that this region contained a thin transition zone and an irregular wavy interface, without noticeable cracks or pores. The wavy interface at position II had an amplitude of approximately 2–6 μm based on SEM measurements. Such an interface morphology appears to be indicative of good bonding quality, as it combines mechanical interlocking from the wavy structure with metallurgical bonding. Position III is situated at the end of the welded zone. As shown in Figure 11d,g,j, the interface in this region was relatively flat and straight, with only a narrow transition layer of approximately 2–5 μm in thickness and no distinct wavy structure, suggesting that severe plastic deformation did not occur here.

**Figure 12:**
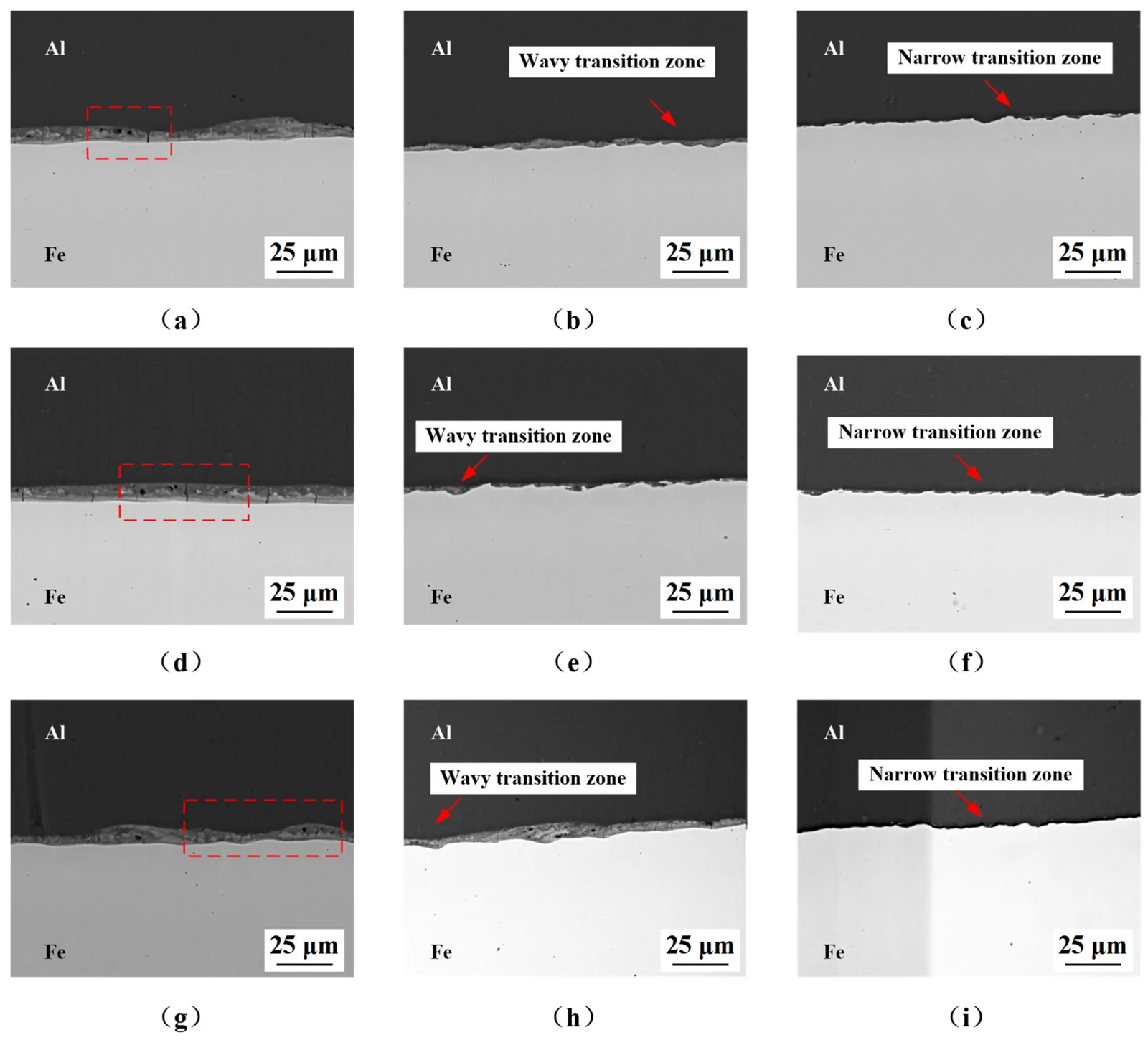
SEM images of the interface at (**a**,**d**,**g**) location I, (**b**,**e**,**h**) location II, and (**c**,**f**,**i**) location III for initial gaps of 1.5 mm, 1.75 mm, and 2.0 mm (discharge voltage: 9 kV). For comparison, panels (**a**–**c**) in this figure are sourced from Figure 11b–d.

Similar interfacial features were observed when the initial gap was varied while keeping the discharge voltage constant at 9 kV. Figure 12a,d,g shows that at position I, cracks and pores were present for all initial gaps (1.5 mm, 1.75 mm, and 2.0 mm), and the defect-containing transition region extended over a width of approximately 4–10 μm. At position II, as seen in Figure 12b,e,h, a relatively continuous transition zone was formed without obvious defects, corresponding to a wavy interface. The wave amplitude was estimated to be approximately 2–7 μm, and the transition zone thickness was about 7 μm. At position III (Figure 12c,f,i), the interface remained flat and the transition zone was narrow, with a thickness of only about 2–5 μm, consistent with the observations under different voltages.

**Figure 13:**
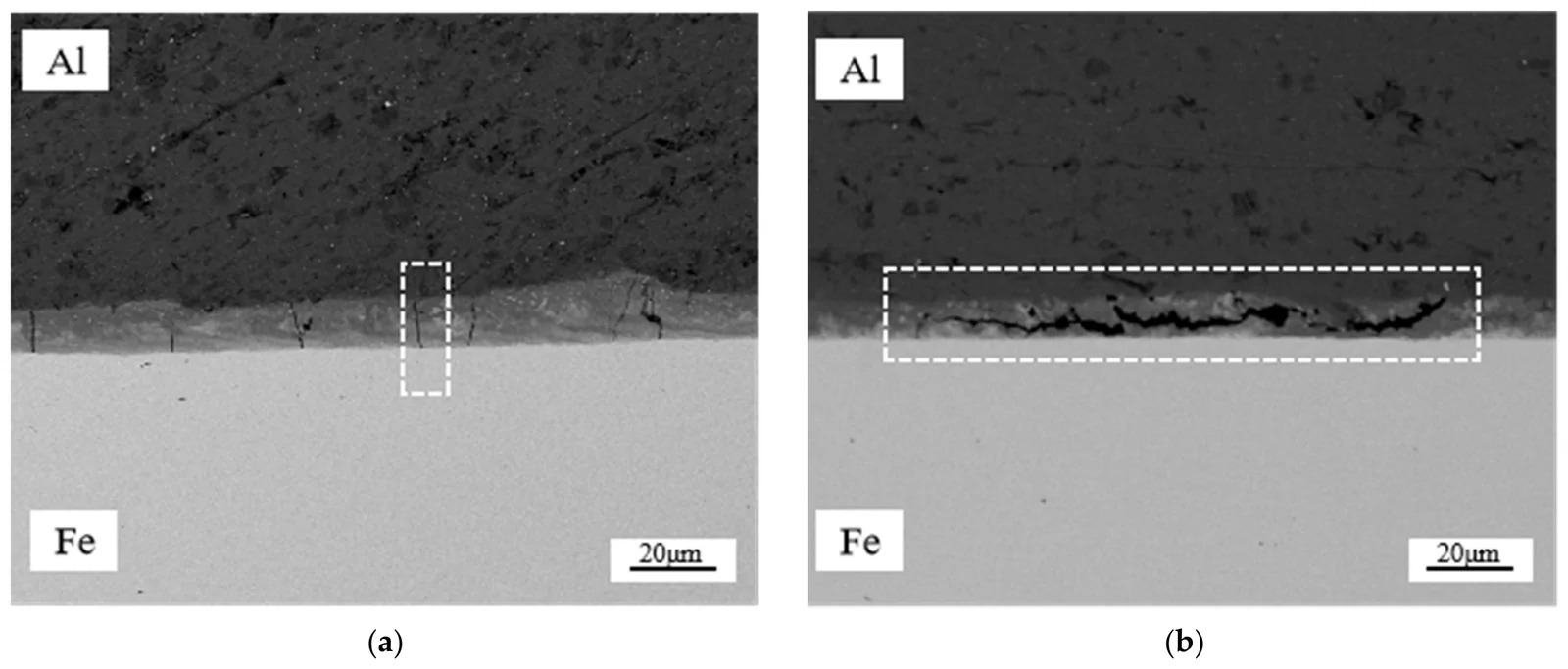
Interfacial micro-crack defects: (**a**) transverse crack in the transition zone and (**b**) longitudinal crack parallel to the weld interface.

In addition to the cracks and pores observed at position I, detailed defect analysis was carried out to understand their formation mechanisms. As shown in Figure 13a, transverse cracks perpendicular to the weld interface were found within the transition zone. This zone appears gray under SEM, which is consistent with previous observations of Fe-Al intermixed regions in impact-welded joints [23]. According to [23], such a gray region is likely formed by rapid solidification of an Fe-Al mixed zone. Rapidly solidified Fe-Al mixed zones have been reported to often contain brittle intermetallic compounds [16,17,19,22]; therefore, the transition zone observed in this study may also contain intermetallic compounds, although direct phase identification was not performed. Because this layer may differ from the adjacent parent metals in thermal and mechanical properties, microcracks may possibly initiate solidification. However, direct evidence of the timing and mechanism of crack initiation was not obtained in this study, and this explanation should be regarded as a possible interpretation rather than a confirmed result.

Figure 13b shows a longitudinal crack parallel to the weld interface within the same transition zone. One possible explanation for this type of crack is stress-wave reflection during impact [37]. According to [37], the compressive wave generated by the collision may be reflected at boundaries and converted into a tensile wave, which could pull apart the intermediate layer if it has not fully solidified. However, this mechanism is also speculative at this stage, and further evidence, such as in situ observation would be required to confirm it. Regardless of the exact formation mechanism, the presence of such defects at position I is consistent with the observation that this region is typically weak and that failure often initiates near the center of the joint when the bonding is insufficient.

Overall, the interfacial morphology under the present welding conditions can be summarized as follows: a central region with cracks and pores, an intermediate region with a wavy interface and a transition zone, and an outer region with a flat interface and a narrow transition layer. Such a wavy interface with a thin transition zone appears to be beneficial for achieving a high-quality joint.

#### 3.4.2. Interfacial Element Intermixing

**Figure 14:**
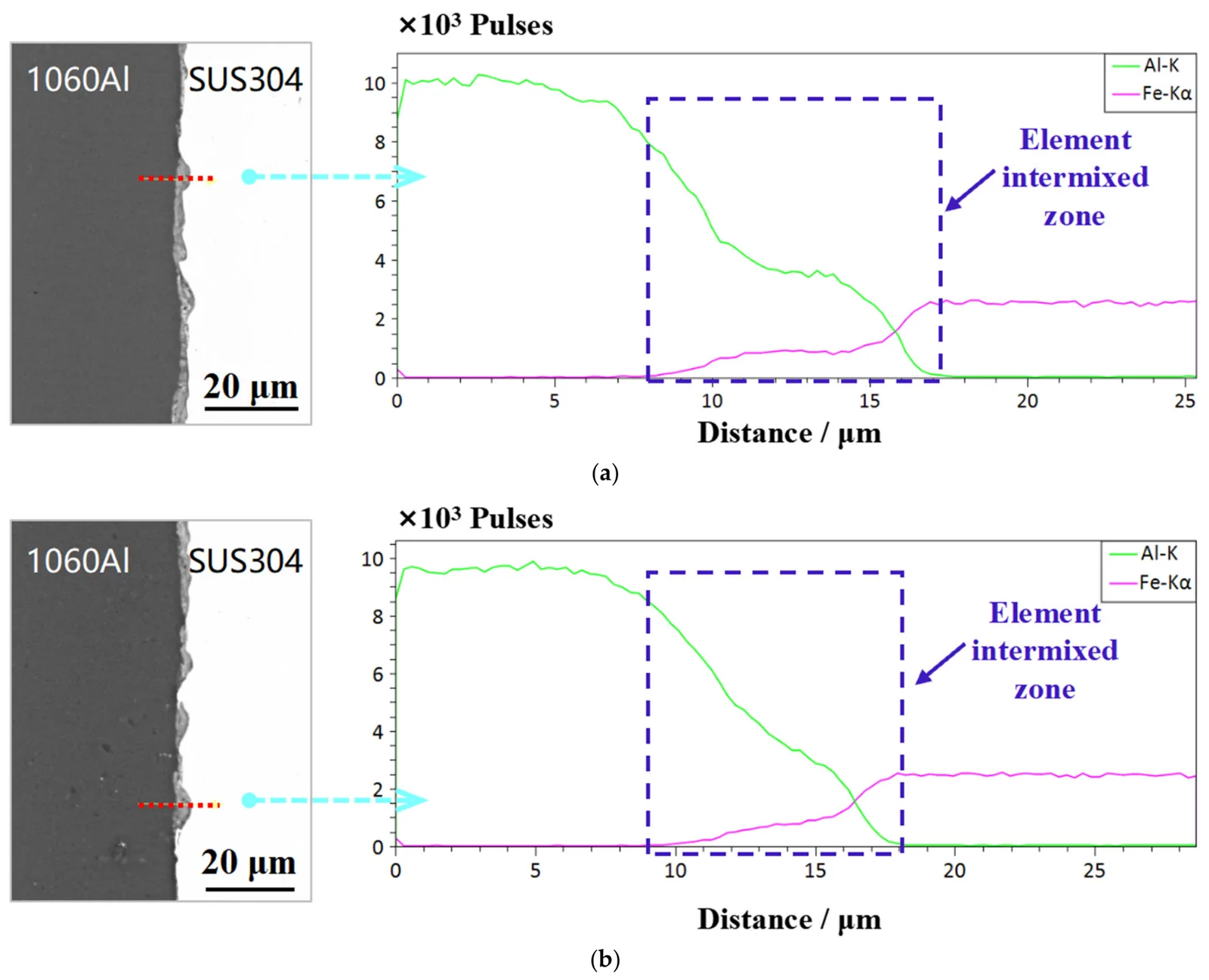
EDS line scan results across typical interfaces at discharge voltages of (**a**) 9 kV and (**b**) 11 kV, showing the apparent width of the intermixed transition zone. The measured width should be considered an apparent value due to the limited spatial resolution of EDS.

To further investigate the metallurgical bonding characteristics, EDS line scans were performed across the interface of typical joints welded at different discharge voltages. Figure 14 shows the results for joints obtained at 9 kV and 11 kV. In this study, the region where Al and Fe intermixing occurs is defined as the transition zone. Under SEM observation, this zone appears gray in contrast, clearly distinguishable from the aluminum side (light) and the steel side (dark). It should be noted that the presence of the transition zone is not necessarily related to whether the interface is wavy or flat.

At a discharge voltage of 9 kV, the EDS line scan in Figure 14a reveals a clear intermixed region at the interface, indicating that Al and Fe are mixed within the transition zone. The apparent width of this region is approximately 7 μm. When the discharge voltage is increased to 11 kV, as shown in Figure 14b, the apparent width increases to approximately 10 μm. It should be noted that the spatial resolution and interaction volume of EDS may cause the measured width to overestimate the true thickness of the intermixed layer. Therefore, the values reported here should be considered as apparent widths of the intermixed zone rather than the actual thickness of any intermetallic layer. Nevertheless, the relative trend suggests that a higher discharge voltage is associated with more extensive interfacial intermixing, which may be related to the higher discharge energy and the associated increase in local temperature and plastic deformation.

It should be noted that MPW is an extremely rapid process, and the high temperature at the interface is maintained only for an extremely short duration. Therefore, the formation of the transition zone is unlikely to be attributed to prolonged solid-state diffusion. Instead, previous studies on MPW and explosive welding have reported that localized melting and rapid solidification are considered to be the main mechanisms for interface bonding in dissimilar metal systems [16,21,38,39,40,41]. According to these studies, the temperature at the interface can exceed the melting points of both Al and Fe under extremely high pressure, leading to the formation of a local melting zone where the two materials are mixed in the molten state. The molten pool then undergoes rapid solidification at a cooling rate of 10^7^–10^9^ K/s [42], resulting in the formation of the transition zone observed in this study. Within this rapidly solidified zone, limited atomic interdiffusion may still occur, but it is more likely to occur in the molten state and during the subsequent quenching process rather than being governed by solid-state diffusion.

The apparent width of the transition zone can serve as a semi-quantitative indicator of interfacial intermixing. However, a wider transition zone is not necessarily beneficial, as it may be associated with more solidification defects, such as micro-cracks. In contrast, a thin transition zone appears to be favorable for achieving good bonding, especially in wavy interface regions. The observed variation in transition zone width with process parameters suggests that both discharge voltage and initial gap play a role in controlling the interfacial microstructure and, consequently, the mechanical properties of the joints.

#### 3.4.3. Hardness and Strengthening Mechanisms of Wavy and Straight Interfaces

Based on the interfacial morphology observations in Section 3.4.1, it is clear that wavy interfaces are typically associated with a thin transition zone, whereas straight interfaces tend to exhibit little or no transition zone. To evaluate the local mechanical response of these two interface types and to clarify their distinct strengthening mechanisms, microhardness measurements were conducted across representative interfaces with and without a transition zone. It should be noted that the spacing between adjacent indentations may not fully satisfy the standard requirement for soft materials, and the measured hardness values should therefore be regarded as a qualitative indication rather than a quantitative measure.

**Figure 15:**
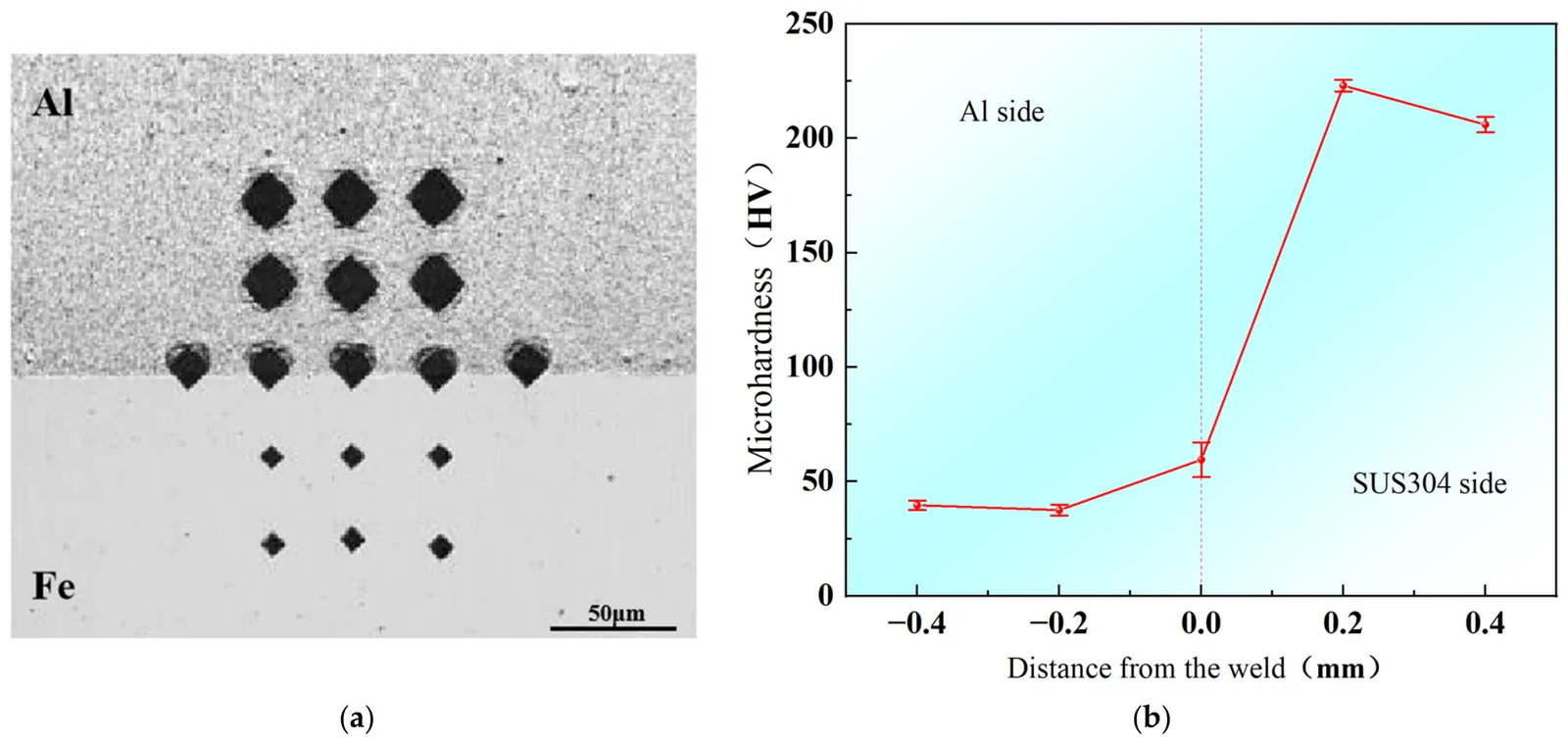
Microhardness measurement results for a straight interface without a transition zone: (**a**) indentation positions and (**b**) hardness distribution. The indentation spacing may not fully satisfy the standard requirement, and the data are presented as a qualitative indication only.

Figure 15 shows the hardness profile across a straight interface without a transition zone. The hardness near the interface was only slightly higher than that of the AA1060 base material and remained lower than that of the SUS304 base material. Since no obvious intermediate layer was formed at this interface, the slight increase in hardness may reflect local microstructural changes near the interface [42,43]. However, given the small magnitude of this increase and the possible influence of insufficient indentation spacing, this result is considered a qualitative trend only. A more precise characterization of the local hardness within such an extremely thin interfacial region would require nanoindentation, which is beyond the scope of the present work and will be addressed in future studies.

**Figure 16:**
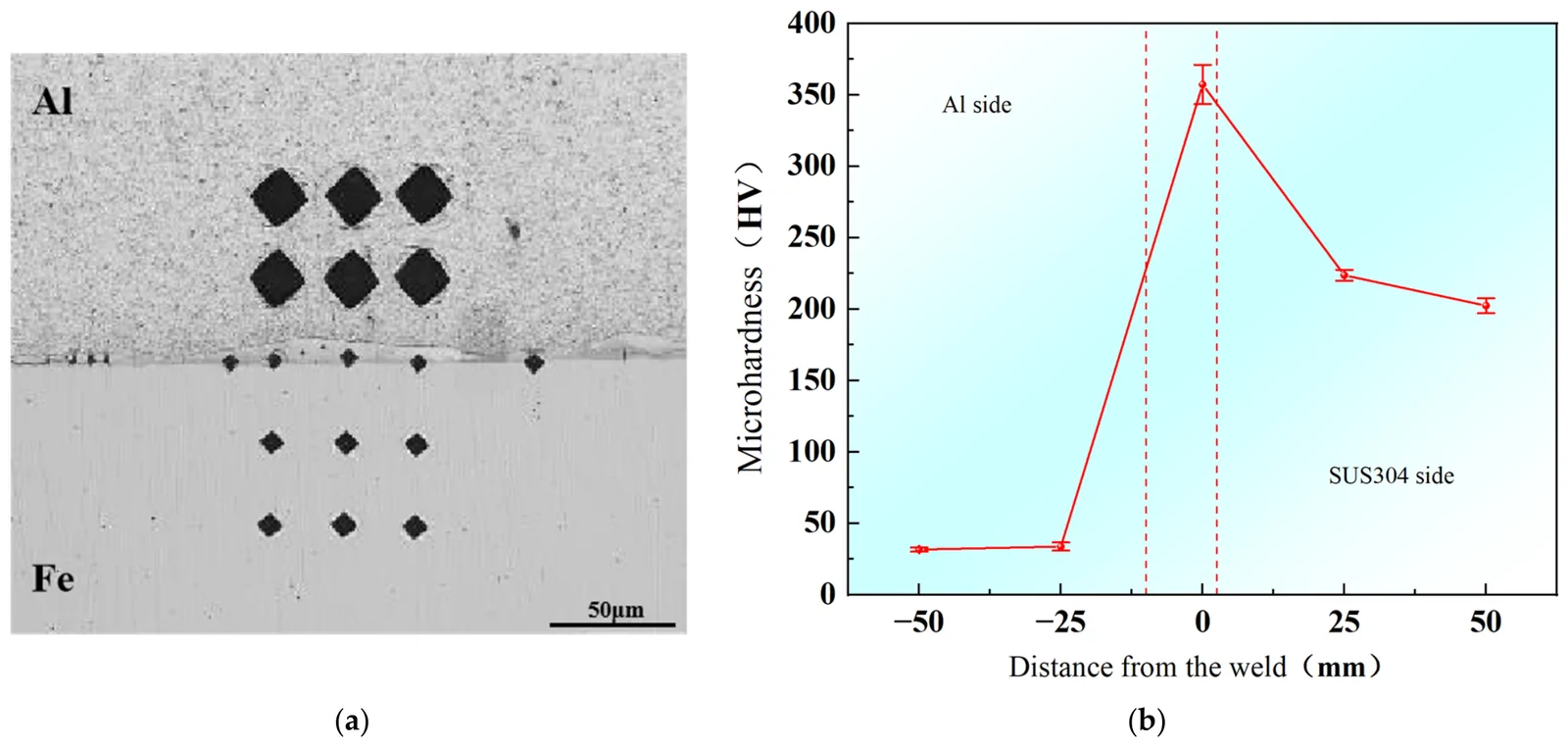
Microhardness measurement results for a wavy interface with a transition zone: (**a**) indentation positions and (**b**) hardness distribution. The indentation spacing may not fully satisfy the standard requirement, and the data are presented as a qualitative indication only.

In contrast, Figure 16 presents the hardness distribution across a wavy interface with a transition zone. The hardness within the transition zone was markedly higher than that of both base materials. This pronounced hardening may be associated with the formation of a rapidly solidified Fe-Al intermixed zone, which possibly contains hard intermetallic compounds and is considerably harder than both parent materials [16,27]. Although local plastic deformation and impact-induced refinement may also occur near the interface during the high-velocity collision, the main contribution to the pronounced hardness increase within the transition zone appears to be the rapidly solidified Fe-Al intermixed structure.

These results suggest that wavy interfaces with a thin transition zone combine mechanical interlocking with metallurgical bonding, resulting in higher local hardness and joint performance, whereas straight interfaces without a transition zone show lower hardness. Under the parameters giving the highest performance within the investigated range (9 kV discharge voltage and 1.5 mm initial gap), the welded zone was dominated by wavy interfaces with thin transition zones, while the defect-containing central region remained limited. This favorable combination increased the effective bonded area and contributed to a mean maximum tensile–shear load of 3288 ± 256 N, with failure in the aluminum base material.

It is important to note that the tensile–shear load reflects the overall response of the entire welded joint, not solely the behavior of a single interfacial region. The joint simultaneously contains multiple interfacial morphologies, such as wavy, straight, and defect-containing zones, and the measured load is determined by both the intrinsic strength of each zone and the size of the effectively bonded area. Microhardness measurements provide local mechanical information and confirm that the wavy interface with a transition zone has a higher strengthening capacity than the straight interface or the base materials. However, the improved tensile–shear performance under the parameters giving the highest performance within the investigated range should be understood as a result of the expanded wavy-interface region and suppression of central defects, rather than the existence of a wavy interface alone.

### 3.5. Fracture Morphology and Fracture Mechanism

#### 3.5.1. Fracture Morphology of Base Material Fracture

**Figure 17:**
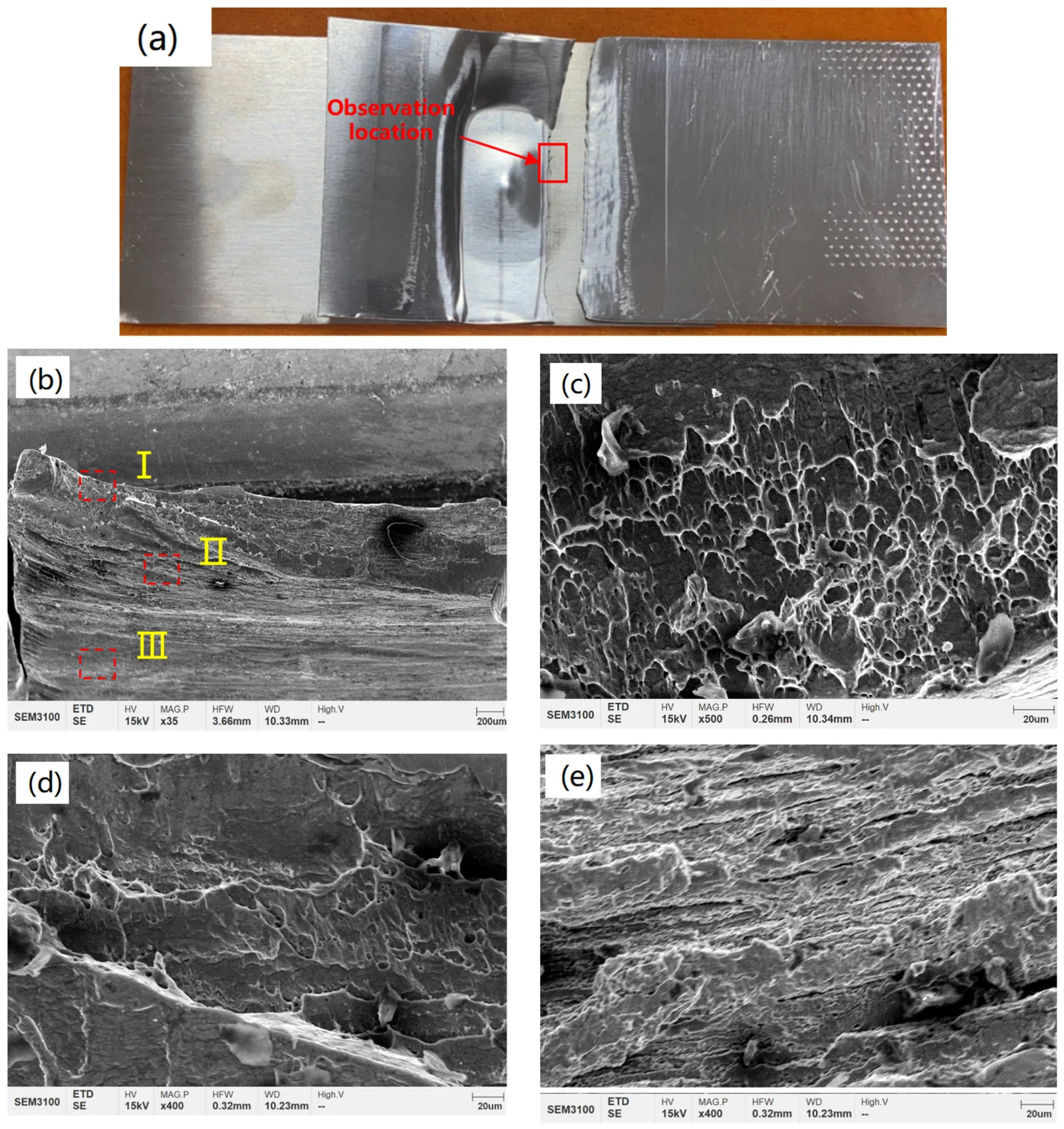
Fracture surface morphology of a specimen that failed in the aluminum base material: (**a**) photograph of the specimen after testing showing the region subjected to fractographic analysis, (**b**) overall macroscopic fracture surface, (**c**) region I (inner side), (**d**) region II (middle), (**e**) region III (outer side).

Figure 17a shows the macroscopic appearance of a tested specimen, with the region selected for fractographic analysis marked. It should be noted that this specimen failed in the aluminum base material, and the fracture path was located close to the weld interface. The fracture surface was examined by SEM. As shown in Figure 17b, the overall fracture surface exhibits distinct morphological differences from the inner side to the outer side. Region I, located at the inner side, is characterized by numerous dimples with irregular shapes and varying sizes and depths (Figure 17c), indicating plastic deformation and a ductile fracture mode. Region II, in the middle of the fracture surface, exhibits a mixed morphology of dimples and river-like patterns (Figure 17d), suggesting that both ductile and brittle fracture occurred. Region III, on the outer side, displays a relatively flat fracture surface with river-like patterns (Figure 17e), which is characteristic of quasi-cleavage fracture.

The transition from ductile to quasi-cleavage fracture across the fracture surface can be attributed to the combined effects of plastic constraint and stress state. In dissimilar joints between materials with a large strength difference, such as aluminum and stainless steel, the deformation of the soft aluminum can be constrained by the adjacent hard stainless steel. This plastic constraint elevates the stress triaxiality near the interface and suppresses plastic deformation of the aluminum, promoting an apparently brittle fracture morphology even though fracture occurs within the aluminum. Similar fracture morphologies have been reported for Al/steel explosive-welded joints, where the fracture surface exhibited ductile and quasi-cleavage features [44,45], and for laser-brazed Al/steel joints, where hard and brittle interfacial intermetallic compounds led to brittle fracture [46]. In the present study, the inner side of the fracture surface, which is farther from the interface and less affected by constraint, exhibits a more ductile mode, whereas the outer side, which experiences stronger constraint, exhibits quasi-cleavage features. Therefore, the overall fracture mode of the base material fracture specimens should be described as a mixed ductile-brittle fracture, consistent with the microstructural observations and the tensile–shear results.

#### 3.5.2. Fracture Morphology of Insufficiently Bonded Joints

**Figure 18:**
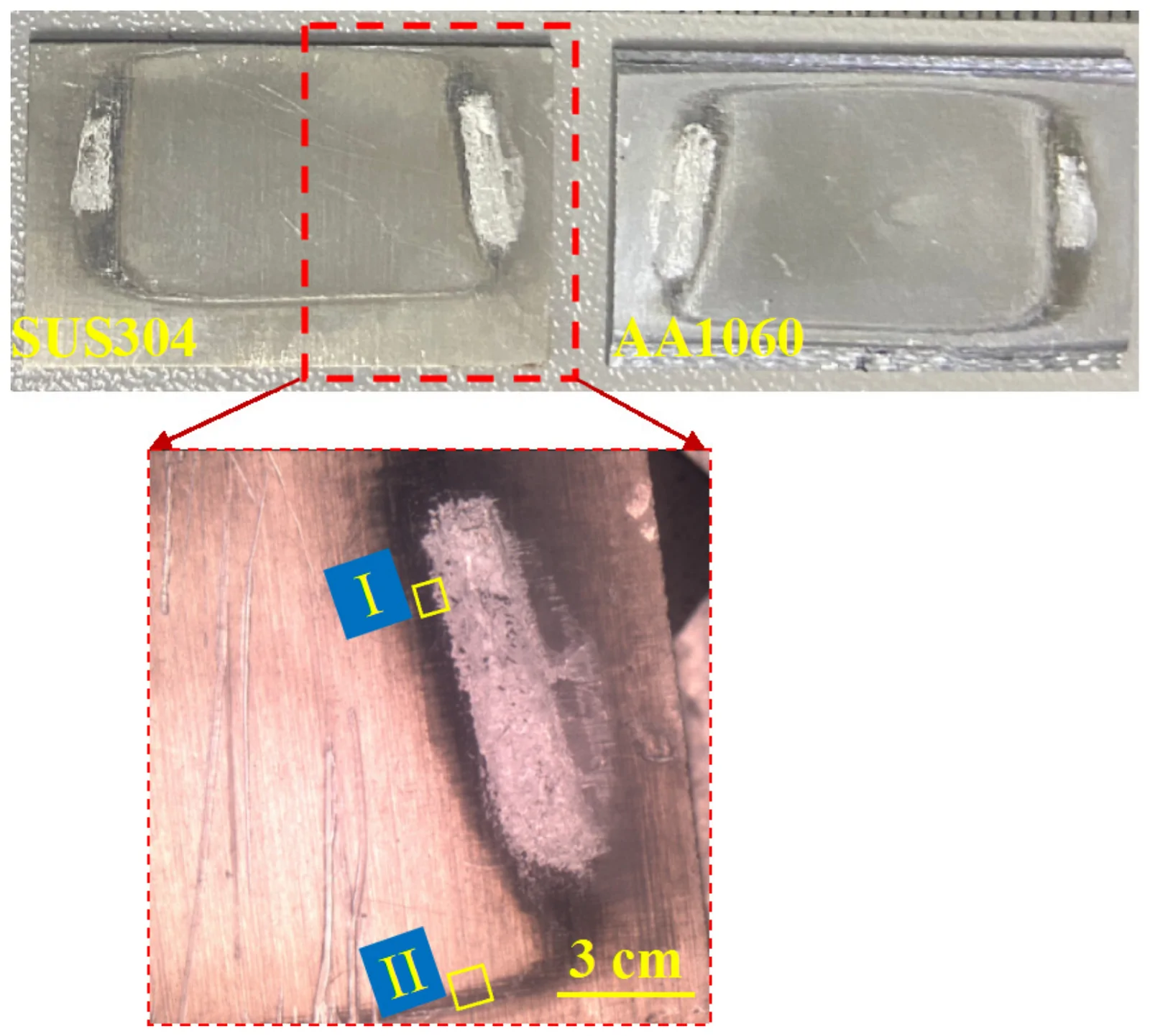
Macroscopic fracture morphology of an insufficiently bonded joint showing selected observation positions I and II.

To understand the bonding condition of joints that failed by pull-out, SEM observation was performed on the fracture surfaces of specimens that exhibited insufficient bonding. Figure 18 shows the macroscopic fracture morphology of such a joint. The weld trace displayed a nearly square annular shape: the short side exhibited a relatively wide weld trace with visible aluminum adhesion on the stainless steel surface, indicating that bonding occurred in this region, whereas the long side showed a narrow and indistinct weld trace, making it difficult to determine whether bonding had occurred. Two typical positions, marked as I (on the short side) and II (on the long side), were selected for further microstructural analysis.

**Figure 19:**
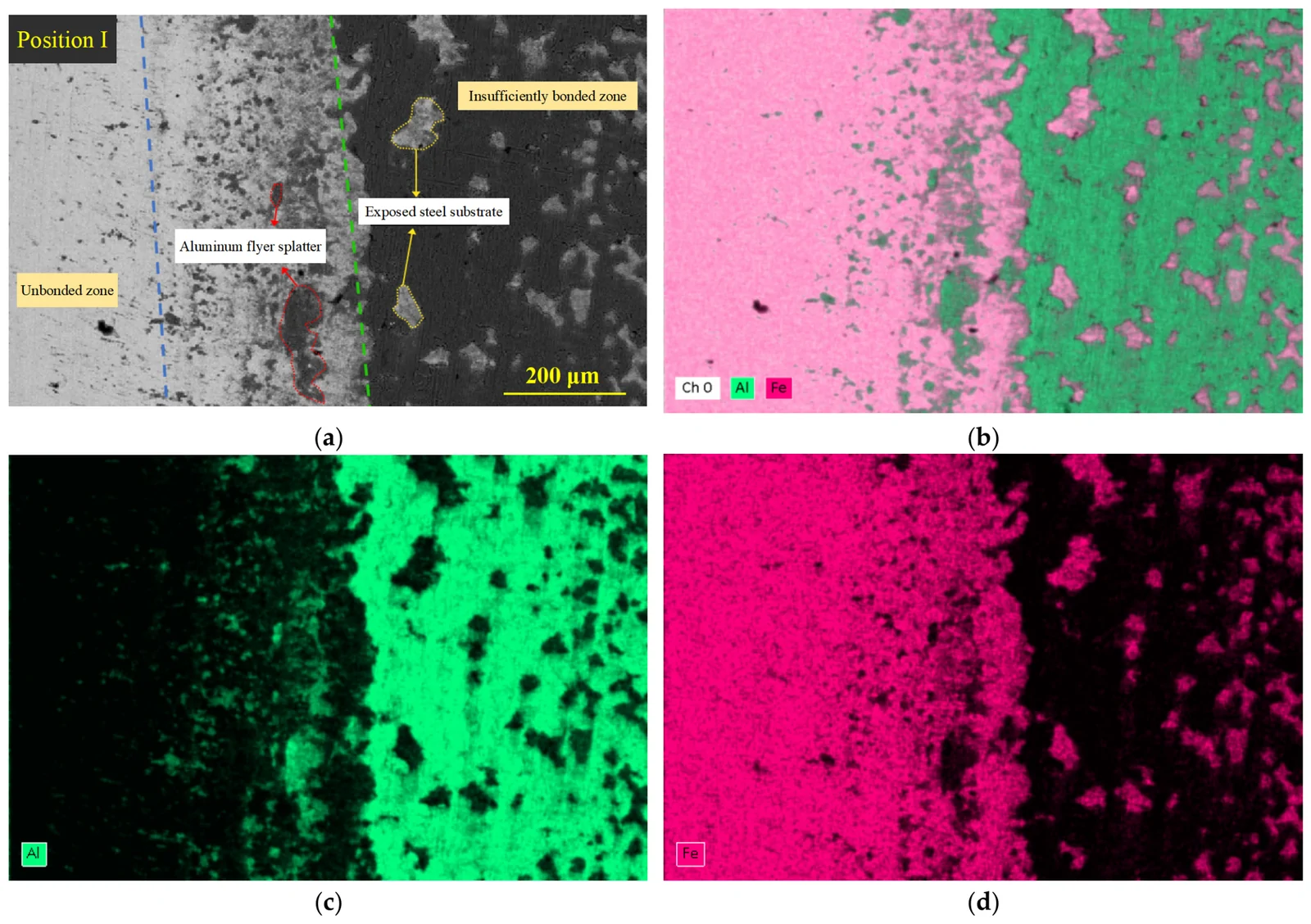
Fracture morphology and EDS elemental maps at position I: (**a**) SEM morphology, (**b**) combined Al-Fe map, (**c**) Al map, (**d**) Fe map.

At position I, as shown in Figure 19a, the fracture surface was dominated by exposed stainless steel substrate. This observation suggests that, although severe collision deformation occurred, an effective collision angle was not achieved, likely resulting in a nearly perpendicular impact and large rebound that prevented the formation of a sound weld. In the insufficiently bonded area, a large amount of bare stainless steel was observed, suggesting that the impact energy was too low to cause sufficient plastic deformation of the aluminum sheet. In addition, dispersed aluminum particles were observed between the unbonded and insufficiently bonded regions. In impact welding processes such as magnetic pulse welding, a metal jet is typically emitted from the collision point during oblique high-velocity impact, and this jet is composed of metallic particles that are expelled from the workpiece surface. Numerical simulations of magnetic pulse welding have also shown that the material ejection predicted during collision explains the experimentally observed deposited particles outside the welded region [47]. Therefore, the dispersed aluminum particles observed in the present study are most likely associated with the material ejected by jetting during the collision [48]. The EDS elemental maps in Figure 19b–d confirm the elemental distribution: the unbonded area is dominated by Fe, while Al is mainly distributed in the bonded region, with only scattered Al particles in the transition area.

**Figure 20:**
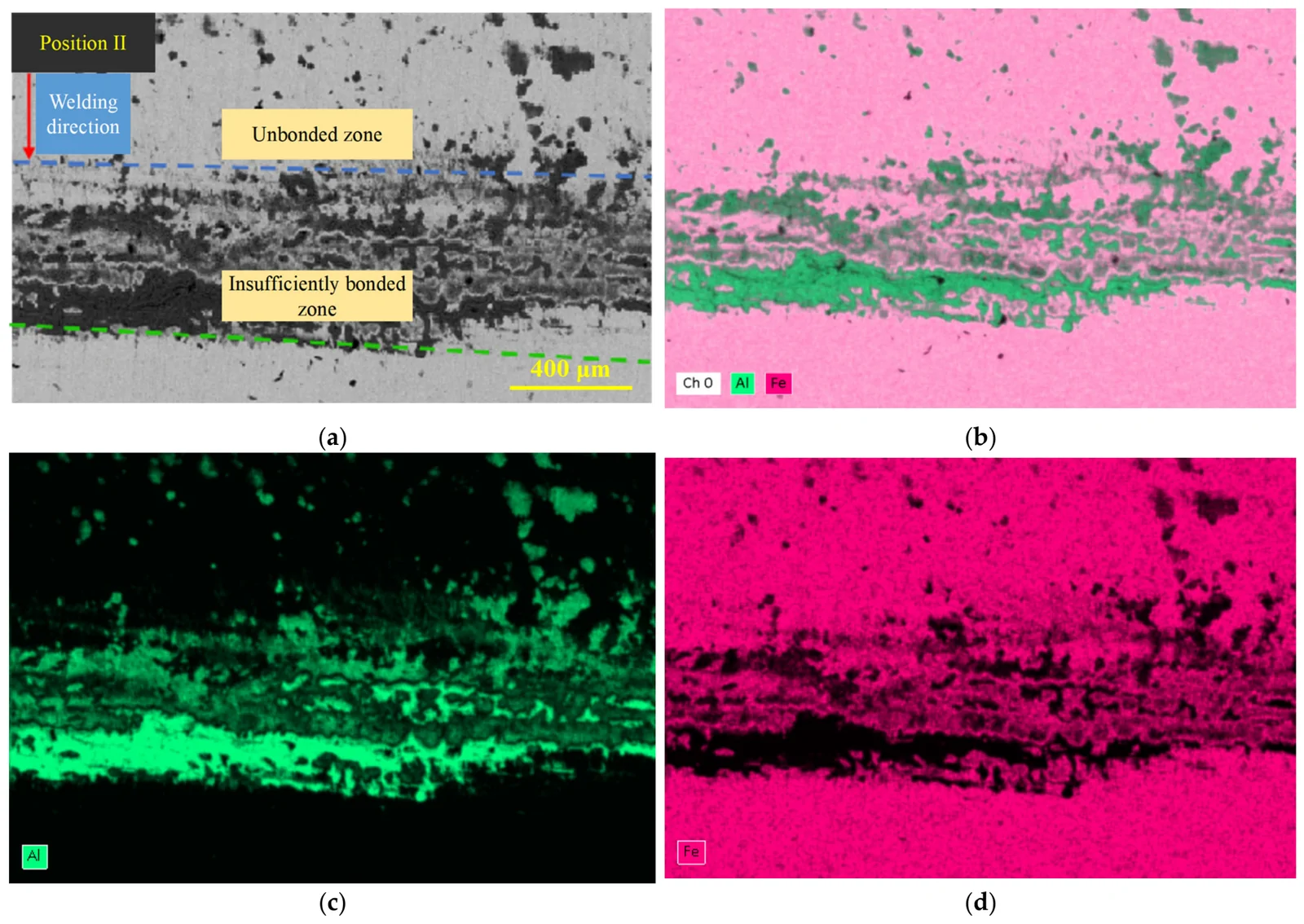
Fracture morphology and EDS elemental maps at position II: (**a**) SEM morphology, (**b**) combined Al-Fe map, (**c**) Al map, (**d**) Fe map.

At position II on the long side, as shown in Figure 20a, the insufficiently bonded zone was narrower than that at position I, and the exposure of the stainless steel substrate was more severe. This suggests that the impact energy at this location was even lower, which likely prevented the two materials from forming an effective bond. The EDS maps in Figure 20b–d further support that very limited Al adhesion occurred on the stainless steel surface at this position.

The fracture morphology of insufficiently bonded joints suggests that the pull-out failure mode is characterized by localized bonding only in certain regions, while other regions remain unbonded or only weakly bonded. This is consistent with the tensile–shear results, where joints failing by pull-out exhibited lower maximum loads and greater variability. The transition from pull-out failure to base material fracture with optimized process parameters reflects an improvement in interfacial bonding uniformity and strength.

## 4. Conclusions

In this work, uniform-pressure electromagnetic pulse welding was employed to join AA1060 aluminum sheets to SUS304 stainless steel sheets. The effects of discharge voltage and initial gap on the interfacial microstructure and mechanical properties of the joints were investigated. The main conclusions are as follows:

(1) The maximum tensile–shear load first increased and then decreased with increasing discharge voltage or initial gap. The highest joint performance within the investigated range was obtained at a discharge voltage of 9 kV and an initial gap of 1.5 mm, with a mean maximum tensile–shear load of 3288 ± 256 N and failure in the aluminum base material. The preliminary process window suggests that discharge voltage and initial gap should be appropriately matched; excessive voltage or gap appeared to cause flyer thinning and reduce joint strength.
(2) The weld interface consisted of three typical zones: a central zone with cracks and pores, an intermediate zone with a wavy interface and a transition zone, and an outer zone with a straight interface and a narrow transition layer. Under the parameters giving the highest performance within the investigated range, the wavy interface with a thin transition zone dominated the welded area, while the defect-containing central zone remained limited, thereby increasing the effective bonded area and contributing to failure in the base material. The apparent width of the transition zone increased with discharge voltage, but a wider transition zone was not necessarily beneficial, as it may be associated with more solidification defects.
(3) Microhardness measurements showed that the straight interface without a transition zone exhibited hardness values between those of the base materials, whereas the wavy interface with a transition zone exhibited higher hardness than both parent materials, suggesting stronger local strengthening at the wavy interface. This pronounced hardening may involve the rapidly solidified Fe-Al intermixing zone. However, direct phase identification within the Fe-Al intermixing zone was not available in this study.
(4) Fracture surface analysis showed that base material fracture was characterized by mixed ductile and brittle features, whereas pull-out fracture exhibited localized bonding and exposed stainless steel substrate. The transition from pull-out failure to base material fracture with optimized process parameters suggests improved interfacial bonding uniformity and strength.

## Figures and Tables

**Table 1:** Chemical composition of AA1060 aluminum alloy (wt.%).

Element	Mn	Si	Mg	Cu	Zn	Fe	Al
Content	0.01	0.03	0.03	0.03	0.05	0.15	Bal.

**Table 2:** Chemical composition of SUS304 stainless steel (wt.%).

Element	C	Si	Mn	P	S	Cr	Ni	Fe
Content	0.048	0.459	1.081	0.030	0.004	18.180	8.050	Bal.

**Table 3:** Mechanical properties of the test materials.

Material Types	Yield Strength	Tensile Strength	Elongation
AA1060-O	30 MPa	95 MPa	34%
SUS304-O	265 MPa	650 MPa	56%

**Table 4:** Maximum tensile–shear loads and corresponding failure modes of joints welded at different discharge voltages (initial gap: 1.5 mm).

Discharge Voltage (kV)	Maximum Tensile–Shear Load (N)	Failure Mode
8.0	1580	Joint pull-out
	890	Joint pull-out
	1065	Joint pull-out
9.0	3015	Base material fracture
	3522	Base material fracture
	3328	Base material fracture
10.0	2730	Base material fracture
	2872	Base material fracture
	2455	Base material fracture
11.0	1984	Base material fracture
	2625	Base material fracture
	2487	Base material fracture

**Table 5:** Maximum tensile–shear loads and corresponding failure modes of joints welded at different initial gaps (discharge voltage: 9.0 kV).

Initial Gap (mm)	Maximum Tensile–Shear Load (N)	Failure Mode
1.0	1289	Joint pull-out
	2130	Joint pull-out
	1058	Joint pull-out
1.25	2325	Joint pull-out
	2455	Joint pull-out
	1780	Joint pull-out
1.5	3015	Base material fracture
	3522	Base material fracture
	3328	Base material fracture
1.75	2950	Base material fracture
	2705	Base material fracture
	3100	Base material fracture
2.0	3019	Base material fracture
	2456	Base material fracture
	2963	Base material fracture

## Data Availability

The original contributions presented in this study are included in the article. Further inquiries can be directed to the corresponding author.
